# Transcriptomics‐Based Liquid Biopsy for Early Detection of Recurrence in Locally Advanced Gastric Cancer

**DOI:** 10.1002/advs.202406276

**Published:** 2024-11-18

**Authors:** Ping'an Ding, Jiaxiang Wu, Haotian Wu, Tongkun Li, Xiaoman Niu, Peigang Yang, Honghai Guo, Yuan Tian, Jinchen He, Jiaxuan Yang, Renjun Gu, Lilong Zhang, Ning Meng, Xiaolong Li, Zhenjiang Guo, Lingjiao Meng, Qun Zhao

**Affiliations:** ^1^ The Third Department of Surgery the Fourth Hospital of Hebei Medical University Shijiazhuang 050011 China; ^2^ Hebei Key Laboratory of Precision Diagnosis and Comprehensive Treatment of Gastric Cancer Shijiazhuang 050011 China; ^3^ Big data analysis and mining application for precise diagnosis and treatment of gastric cancer Hebei Provincial Engineering Research Center Shijiazhuang 050011 China; ^4^ School of Chinese Medicine & School of Integrated Chinese and Western Medicine Nanjing University of Chinese Medicine Nanjing Jiangsu 210023 China; ^5^ Department of Gastroenterology and Hepatology Jinling Hospital Medical School of Nanjing University Nanjing Jiangsu 210002 China; ^6^ Department of General Surgery Renmin Hospital of Wuhan University Wuhan Hubei 430065 China; ^7^ Department of General Surgery Shijiazhuang People's Hospital Shijiazhuang Hebei 050050 China; ^8^ Department of General Surgery Baoding Central Hospital Baoding Hebei 071030 China; ^9^ Department of General Surgery Hengshui People's Hospital Hengshui Hebei 053099 China; ^10^ Research Center and Tumor Research Institute of the Fourth Hospital of Hebei Medical University Shijiazhuang 050011 China

**Keywords:** gastric cancer, liquid biopsy, mRNA Panel, recurrence detection, transcriptomics

## Abstract

The study presents a transcriptomics‐based liquid biopsy approach for early recurrence detection in locally advanced gastric cancer (LAGC). Four mRNA biomarkers (AGTR1, DNER, EPHA7, and SUSD5) linked to recurrence are identified through transcriptomic data analysis. A Risk Stratification Assessment (RSA) model combining these biomarkers with clinical features showed superior predictive accuracy for postoperative recurrence, with AUCs of 0.919 and 0.935 in surgical and liquid biopsy validation cohorts, respectively. Functional studies using human gastric cancer cell lines AGS and HGC‐27 demonstrated that silencing the identified mRNA panel genes impaired cell migration, invasion, and proliferation. In vivo experiments further showed reduced tumor growth, metastasis, and lymphangiogenesis in mice, possibly mediated by the cAMP signaling pathway. This non‐invasive approach offers significant potential for enhancing recurrence detection and enabling personalized treatment strategies, thereby improving patient outcomes in the management of LAGC.

## Graphical abstract

A novel liquid biopsy method using a 4‐mRNA biomarker panel (AGTR1, DNER, EPHA7, SUSD5) improves early detection of recurrence in locally advanced gastric cancer. A risk stratification model incorporating this panel and clinical features shows superior accuracy, reducing unnecessary interventions and enabling personalized treatment strategies to improve patient outcomes.

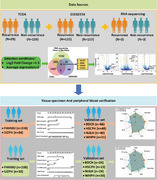

## 1 Introduction

Gastric cancer remains a major global health challenge, ranking as the fifth most common malignancy and the third leading cause of cancer‐related mortality.^[^ ^1^, ^2^, ^3^ ^]^ Patients diagnosed with locally advanced gastric cancer (LAGC) have a particularly poor prognoses, primarily due to the high rates of recurrence and metastasis.^[^ ^4^, ^5^ ^]^ Despite improvements in treatment strategies, including surgical resection, chemotherapy, and targeted therapies, the overall survival rate for patients with LAGC remains suboptimal, with approximately 50–60% of LAGC patients experiencing recurrence within two years of initial treatment.^[^ ^6^, ^7^, ^8^, ^9^ ^]^ Early detection of this recurrence is crucial as it has a significant impact on patients' survival and quality of life.^[^ ^10^ ^]^ Recurrence often signifies a progression to a more advanced disease state, which is harder to treat effectively.

Current diagnostic methods for detecting recurrence in LAGC include imaging techniques such as computed tomography (CT) scans,^[^ ^11^, ^12^ ^]^ magnetic resonance imaging (MRI) scans,^[^ ^13^, ^14^ ^]^ and positron emission tomography (PET) scans,^[^ ^15^, ^16^, ^17^ ^]^ along with endoscopic evaluations.^[^ ^18^, ^19^ ^]^ While these methods have contributed to the management of gastric cancer, they have inherent limitations. First, imaging modalities are often insufficiently sensitive to detect early‐stage or occult metastases, leading to delayed diagnosis and reduced treatment efficacy.^[^ ^20^ ^]^ Second, these techniques are invasive, expensive, and may expose patients to radiation or procedural risks.^[^ ^21^ ^]^ Furthermore, they are typically performed at scheduled intervals, which may miss the window of early molecular recurrence, thus delaying potential interventions.^[^ ^22^ ^]^

The development of non‐invasive, sensitive, and specific diagnostic tools for early detection of gastric cancer recurrence has become an active area of research. One of the most promising alternatives to traditional diagnostic methods is liquid biopsy.^[^ ^23^, ^24^, ^25^ ^]^ Liquid biopsy refers to the analysis of non‐solid biological tissue, primarily blood, to detect cancer‐related biomarkers such as circulating tumor cells (CTCs),^[^ ^26^, ^27^, ^28^ ^]^ cell‐free DNA (cfDNA),^[^ ^29^, ^30^, ^31^, ^32^ ^]^ and extracellular vesicles (EVs).^[^ ^33^, ^34^, ^35^, ^36^ ^]^ Despite the promising potential, the traditional components of liquid biopsy, such as cfDNA and CTCs, still face challenges in sensitivity and specificity. ^[^ ^37^ ^]^ Herein lies the potential of transcriptomics, the study of RNA transcripts, which offers a more dynamic and comprehensive understanding of gene expression changes associated with cancer recurrence. RNA molecules, including messenger RNA (mRNA), microRNA (miRNA), and long non‐coding RNA (lncRNA), reflect the real‐time activity of tumor cells and can provide valuable insights into the molecular underpinnings of recurrence.^[^ ^38^, ^39^, ^40^, ^41^ ^]^

Through systematic and comprehensive transcriptome analysis, we identified a set of novel mRNAs for predicting postoperative recurrence in LAGC patients, which were hypothesized to have good predictive performance in both tissue and liquid biopsy samples and were validated sequentially. The clinical significance of this study lies in its potential to revolutionize the detection and management of gastric cancer recurrence. By developing a transcriptomics‐based liquid biopsy panel, this research aims to offer a non‐invasive, highly sensitive, and specific method for early detection of recurrence in LAGC patients. Such a diagnostic tool could enable continuous monitoring of patients, allowing for earlier interventions and personalized treatment adjustments. In addition, this model will also provide a more convenient way to detect recurrent patients during clinical treatment, allowing patients to benefit more. This approach not only promises to improve patient outcomes by catching recurrences at a stage where they are more treatable but also reduces the physical and financial burdens associated with traditional diagnostic methods. Ultimately, this research could pave the way for broader applications of transcriptomics‐based diagnostics in other cancers, contributing to the advancement of personalized medicine and improving overall cancer care.

## 2 Experimental Section

### 2.1 Comprehensive mRNA Biomarker Discovery

**Figure 1:**
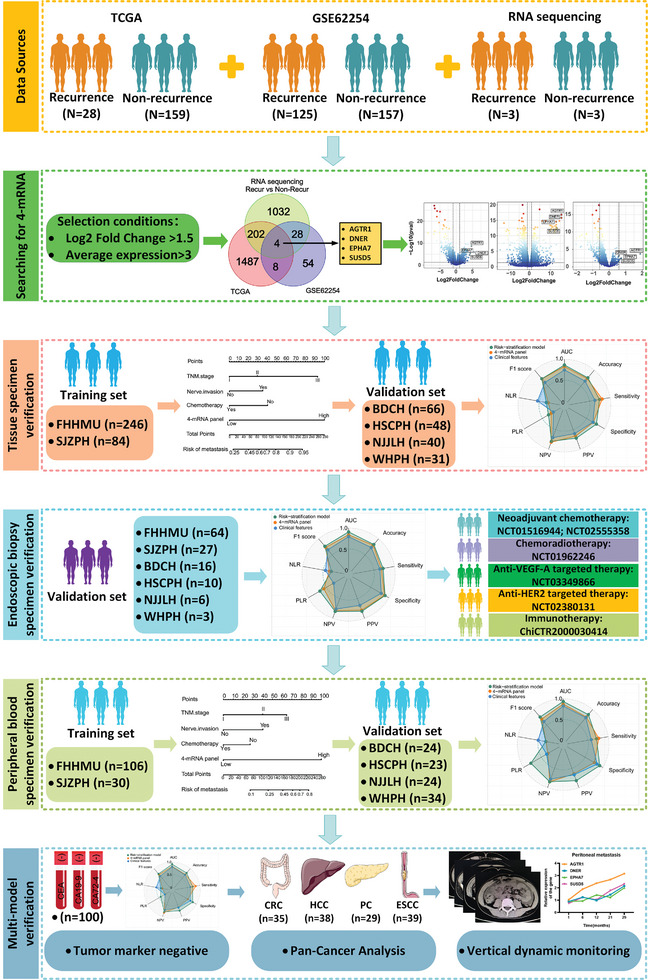
Flowchart of study design for the discovery and validation of the 4‐mRNA panel to predict postoperative recurrence in patients with LAGC.

The biomarker discovery and validation process used in this study is illustrated in **Figure** **1**. Initially, mRNA sequencing data from multiple sources were utilized, including a recurrence cohort from the Gene Expression Omnibus (GEO) database (GSE62254, 125 patients with recurrence vs 157 patients without recurrence), the Cancer Genome Atlas (TCGA) database cohort (28 patients with recurrence vs 159 patients without recurrence), and 6 LAGC patients with cancer tissue specimens (3 recurrent vs 3 non‐recurrent) for biomarker identification.

### 2.2 RNA Extraction and Gene Expression Analysis

For further validation of clinical application performance, a cohort of fresh frozen postoperative specimens, gastroscopic biopsy specimens, and peripheral blood samples from multiple clinical centers were used to ensure thorough and repetitive validation.

Total RNA was isolated from fresh‐frozen surgical tissues using the TRIzol reagent (Invitrogen, Frederick, MA, USA) according to the manufacturer's protocol.^[^ ^42^ ^]^ Take part of the tissue and add 1 mL TRIzol reagent, place it on ice and grind thoroughly. Subsequently add 1/5 volume of chloroform to the mixture, mix by inverting upside down, and place the sample on ice for 5 min. Centrifuge at 12 000 rpm at high speed, 4 °C for 15 min. After 15 min, absorb the supernatant and add an equal volume of isopropyl alcohol. Mix by inverting and let stand on ice for 10 min. Centrifuge again at high speed, 12 000 rpm, 4 °C, for 10 min. After discarding the supernatant, add 400 ul of 75% ethanol, centrifuge at 9000 rpm, 4 °C for 5 min, Discard the supernatant and after the precipitate was dried, add an appropriate amount of DEPC water to dissolve the precipitate.

For serum samples, the PAXgene Blood RNA Kit (Qiagen, Hilden, Germany) was utilized for total RNA extraction.^[^ ^43^ ^]^ The process begins by incubating the resuspended pellet with proteinase K in an optimized buffer for proteolysis. Then use a PAXgene Shredder spin column to centrifuge the homogenized cell lysate again and remove the remaining cell debris. Transfer the flow‐through supernatant to a clean microcentrifuge tube, add ethanol to adjust the binding conditions, and load the lysate into a PAXgene RNA spin column. After a quick centrifugation, RNA was selectively bound to the PAXgene silica membrane and impurities flow through. The remaining impurities were removed through several efficient washes. Between the first and second wash steps, treat the membrane with DNase to remove traces of residual DNA. After washing, RNA was eluted using an elution buffer and thermally denatured.

Subsequently, the GoScript Reverse Transcription System Kit (Promega) was employed to reverse transcribe the total RNA into cDNA following the manufacturer's instructions. Quantitative real‐time polymerase chain reaction (qRT‐PCR) analysis was then performed.^[^ ^44^ ^]^ The relative abundance of target genes was determined using the 2^^−ΔΔCT^ method, normalized to the GAPDH internal control, where ΔCT represents the difference between the target and GAPDH CT values. The specific PCR primers used are listed in Table S6 (Supporting Informmation).

The expression of selected mRNAs was initially assessed using qRT‐PCR in 34 pairs of recurrent and non‐recurrent cancer tissue samples matched by a 1:1 propensity score (Figure S1A; Table S1, Supporting Informmation). Additionally, peripheral blood specimens from 26 pairs of recurrent and non‐recurrent patients, matched by a 1:1 propensity score, and peripheral blood samples from 26 healthy individuals undergoing physical examination during the same timeframe were collected to examine the expression of candidate mRNAs in peripheral blood. These samples were collected at the Fourth Hospital of Hebei Medical University (FHHMU) from June to December 2023, and the clinical characteristics of these patients who obtained peripheral blood samples are detailed in Table S2 (Supporting Informmation).

### 2.3 Clinical Cohorts for Biomarker Validation

First, visualized nomograms were developed to predict recurrence after radical surgery in LAGC patients. The training cohort consisted of 330 patients from FHHMU and Shijiazhuang People's Hospital (SJZPH), while the validation cohort included 185 patients from Baoding Central Hospital (BDCH), Hengshui City People's Hospital (HSCPH), Nanjing University Jinling Hospital (NJJLH), and Wuhan University People's Hospital (WHPH). The clinical characteristics of all patients are detailed in Table S3 (Supporting Informmation). Fresh frozen specimens were collected from January 2017 to December 2019. Exclusion criteria included patients who underwent preoperative neoadjuvant chemotherapy, targeted therapy, or immunotherapy, had residual gastric cancer post‐gastrectomy or had concurrent other tumors.

Further analysis involved 126 matched gastroscopic biopsy specimens from LAGC patients across six institutions to validate the transition from large surgical specimens to small gastroscopic biopsies. Detailed clinical characteristics are presented in Table S4 (Supporting Informmation).

To transition the mRNA panel from tissue specimens to noninvasive liquid biopsies, serum samples from LAGC patients collected at the six centers mentioned above were retrospectively analyzed. The training cohort included 136 patients from FHHMU and SJZPH (June 2017 to December 2020), and the validation cohort comprised serum samples from 105 patients (January 2016 to December 2019) from four other institutions. The same inclusion and exclusion criteria were applied for the fresh frozen specimen cohort, with detailed clinicopathologic data shown in Table S5 (Supporting Informmation).

To assess the mRNA panel's predictive value in other tumor types, qRT‐PCR was performed on serum samples from 131 patients with other gastrointestinal malignancies (39 esophageal, 35 colorectal, 38 liver, and 29 pancreatic cancers) collected from FHHMU between 2021 and 2023.

All patients were monitored for recurrence or disease progression using laboratory tests, endoscopy, and abdominopelvic CT, in accordance with gastric cancer treatment guidelines.^[^ ^45^ ^]^ Tissue specimens were immediately frozen in liquid nitrogen and stored at −80 °C. Surgical specimens were processed according to the Chinese Society of Clinical Oncology guidelines. Tumor and lymph node staging was performed according to the 8th edition of the AJCC. All procedures complied with the Declaration of Helsinki, and written informed consent was obtained from all participants, with approval from the institutional review boards of all participating institutions.

### 2.4 Protein–Protein Interaction (PPI) Network Analysis

The STRING database (https://string‐db.org) was utilized to construct the PPI network for Homo sapiens. The four‐gene candidate list underwent gene set and pathway analyses using Enrichr (https://maayanlab.cloud/Enrichr/).^[^ ^46^ ^]^

### 2.5 EdU Assay

AGS and HGC‐27 cells transfected with siRNA were seeded at a density of 2 × 10^5^ cells per well in 12‐well plates and incubated at 37 °C and 5% CO_2_ in a sterile environment for 24 h. Subsequently, the Cell‐Light EdU DNA Cell Proliferation Kit (EdU: 5′Ethynyl‐ 2′‐deoxyuridine; Ribobio, Guangzhou, China) was employed to assess AGS and HGC‐27 cell proliferation according to the manufacturer's protocol. Cell images were acquired using a Zeiss LSM 900 confocal microscope (Zeiss, Germany), and ImageJ version 1.52 was utilized to quantify the percentage of positive cells. The Mann–Whitney U test was used to compare the differences between the groups, and statistical significance was set at a *p* value of <0.05.

### 2.6 CCK‐8 Assay

Following siRNA transfection, AGS and HGC‐27 cells were seeded at a density of 1 × 10^3^ cells per well in 96‐well plates and cultured at 37 °C under sterile conditions. After overnight adherence, 10 uL of CCK‐8 reagent was added to each well. Subsequent to ≈2 h of incubation at 37 °C, absorbance values were measured at 450 nm using a microplate reader (Tecan, USA).

### 2.7 Colony Formation Assay

AGS and HGC‐27 cells transfected with siRNA were seeded into 6‐well plates and incubated at 37 °C for 1–2 weeks. Once colonies became visible to the naked eye, the cells were fixed with 4% paraformaldehyde and stained with a crystal violet solution. Colonies were observed and quantified under a microscope (Leica, Germany).

### 2.8 Wound Healing Assay

Confluent AGS and HGC‐27 cell monolayers transfected with siRNA were scratched using a 10 uL pipette tip. After 24–48 h, the migration distance across the wound was measured microscopically. Initial images at 0 h served as controls to normalize and calculate the relative migration rate.

### 2.9 Transwell Migration and Invasion Assays

Transwell inserts, either uncoated (migration) or Matrigel‐coated (invasion), were used per the manufacturer's instructions. Transfected AGS and HGC‐27 cells (4 × 10^4^) were seeded into the upper chambers and incubated for 24–48 h. Migrated and invaded cells on the lower surface were quantified across five random microscopic fields.

### 2.10 Western Blotting

Transfected AGS cells were lysed in RIPA buffer with 1% PMSF to extract total protein. Denatured proteins were separated via 10% SDS‐PAGE and transferred to PVDF membranes (Seven, Beijing). Membranes were probed with antibodies against AGTR1 (1:1000 dilutions, 25343‐1‐AP, Proteintech, Chicago, USA), DNER (1:1000 dilutions, 24362‐1‐AP, Proteintech, Chicago, USA), EPHA7 (1:1000 dilutions, 66667‐1‐Ig, Proteintech, Chicago, USA), SUSD5 (1:500 dilutions, bs‐7331R, Bioss, Beijing, China), CREB (1:500 dilutions, 381013, Zenbio, Chengdu, China), p‐CREB (1:500 dilutions, 380697, Zenbio, Chengdu, China), and GAPDH (1:10000 dilutions, 10494‐1‐AP, Proteintech, Chicago, USA). Immunoreactive bands were visualized using the ECL Plus reagent (Solarbio).

### 2.11 Immunohistochemistry (IHC)

Tissue sections were deparaffinized at 60 °C for 2 h and treated with xylene. Antigen retrieval was performed using EDTA, followed by quenching of endogenous peroxidase activity with 3% hydrogen peroxide. Sections were then blocked with 5% bovine serum albumin (BSA) before overnight incubation with primary antibodies at 4 °C. The following day, secondary antibodies were applied, and diaminobenzidine (DAB) and hematoxylin were used for antigen detection and nuclear counterstaining, respectively. ImageJ software was utilized to calculate the percentage of positive cells. The scoring criteria were set based on the percentage of positive cells: 1 for ≤25%, 2 for 26–50%, 3 for 51–75%, and 4 for >75%. Concurrently, scores were assigned according to staining intensity: 1 for no staining, 2 for light brown, 3 for obvious brown, and 4 for dark brown. The final IHC score was obtained by multiplying the coverage and staining intensity scores.

### 2.12 Lentiviral Transfection

siRNAs for the four‐panel genes were packaged into lentiviruses to generate lentiviral vectors that stably knocked down AGTR1 (called si‐AGTR1), DNER (called si‐DNER), EPHA7 (called si‐EPHA7), SUSD5 (called si‐SUSD5), or a scrambled control (called si‐NC). AGS cells were seeded in 6‐well plates at 50% confluence and infected with the stably knocked‐down lentiviruses. Puromycin (2µg ml^−1^) was used for selection for 2 weeks to generate stable transduction pools.

### 2.13 Animal Experiments

In vivo experiments were approved by the Institutional Animal Care and Use Committee (IACUC) of the Fourth Affiliated Hospital of Hebei Medical University (IACUC Approval No. 20240727). All BALB/c mice were purchased from SiPeiFu (Beijing) Biotechnology Co., Ltd. To investigate the effects of gene knockdown on tumorigenicity, lymph node metastasis, and peritoneal metastasis, mice were injected subcutaneously, in the footpad, and intraperitoneally with AGS cells transfected with lentiviruses encoding shRNAs targeting specific genes. ≈30 days after cell injection, when a significant difference was observed between the experimental and control groups, the mice were euthanized, and the experimental results were statistically analyzed. Each mouse underwent only one treatment.

### 2.14 Statistical Analysis

Statistical analyses were conducted using IBM SPSS version 23, R version 3.6.3, and GraphPad Prism version 8.0. Univariate and multivariate logistic regression analyses identified significant clinicopathological variables and mRNA classifiers as covariates; variables significant in univariate analysis were included in multivariate regression. In the discovery phase, differential gene expression between the relapsed and non‐relapsed groups was examined using the Wilcoxon rank‐sum test and the Bonferroni correction. During the clinical validation phase, a gene‐based risk score was modeled using logistic regression with backward elimination, and model performance was assessed using receiver operating characteristic (ROC) curves and area under the curve (AUC) values. AUCs were derived from ROC curves using the pROC package in R, and ROC curve comparisons were performed using the DeLong test. The sensitivity, specificity, positive predictive value (PPV), negative predictive value (NPV), precision, and accuracy of the 4‐mRNA biomarker set in all cohorts were determined using the reportROC package, and results were presented in a confusion matrix. The optimal cutoff value for the ROC curve was determined using the Youden index in the pROC package. The Youden index and median risk score were used to stratify individuals into high‐risk or low‐risk groups for predicting recurrence and detecting metastasis. Disease‐free survival (DFS), defined as the time from curative surgery to disease recurrence or patient death due to disease progression, was analyzed using the Kaplan–Meier method, with a 5 year review of patients who were alive and free of recurrence at this milestone. Patients lost to follow‐up without evidence of recurrence within 5 years were assessed at their last visit. Statistical significance was set at a *p* value of <0.05.

### 2.15 Ethics Approval and Consent to Participate

The study protocol was approved by the Ethics Committee of the Fourth Hospital of Hebei Medical University (approval number: 2024KY127), and informed consent was obtained from all the study participants. All authors followed applicable ethical standards to maintain research integrity without duplication, fraud, or plagiarism.

### 2.16 Availability of Data and Materials

The processed data in this study have been deposited in GEO under accession number GSE248612. The participant data with identifiers used to support the findings of this study were supplied by Qun Zhao under license and thus cannot be made freely available. The requests for access to these data should be made to Qun Zhao, zhaoqun@hebmu.edu.cn.

### 2.17 Ethical Statement

All authors certify that they comply with the ethical guidelines for authorship.

## 3 Results

### 3.1 Identification of Candidate mRNAs Associated with Recurrence in LAGC Patients

**Figure 2:**
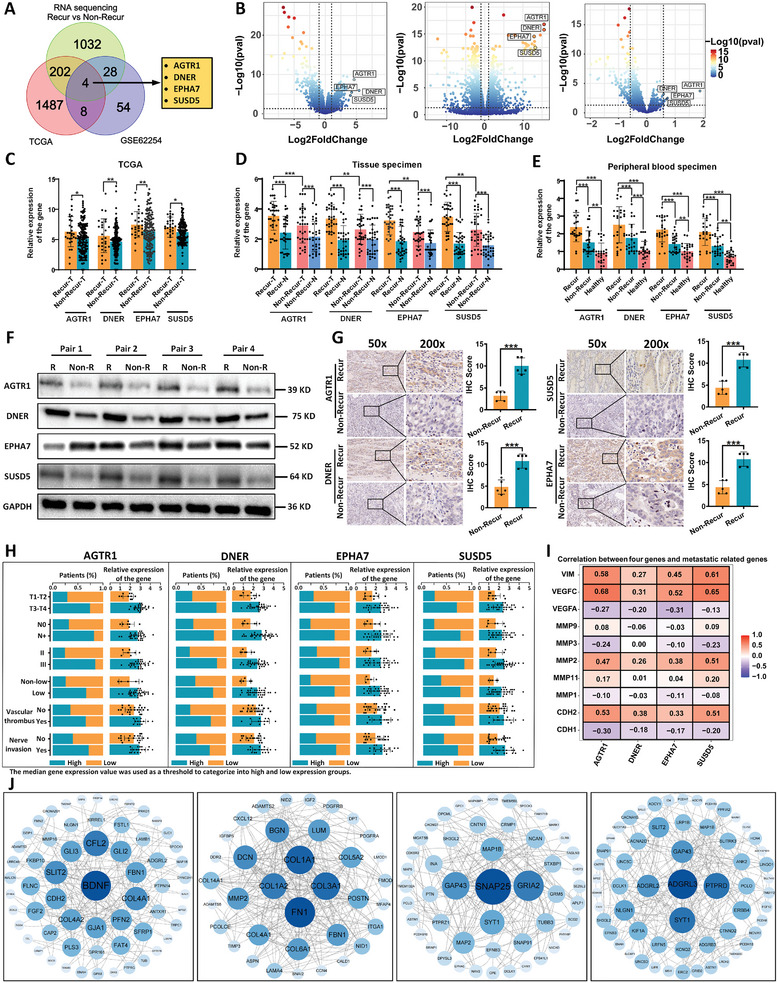
Discovery process and preliminary validation of candidate markers for postoperative recurrence in LAGC patients based on public databases and transcriptomics sequencing data. A) Four candidate mRNAs (AGTR1, DNER, EPHA7, SUSD5) were identified through a Venn diagram analysis using the TCGA database (28 recurrent patients versus 159 non‐recurrent patients), transcriptome data from the GEO database (125 recurrent patients versus 157 non‐recurrent patients), and paired mRNA sequencing (3 recurrent patients versus 3 non‐recurrent patients). B) A volcano plot illustrates the expression levels of these four genes in recurrent and non‐recurrent cancer tissues. C) The expression levels of the four candidate mRNAs (AGTR1, DNER, EPHA7, SUSD5) were compared in cancerous lesions of recurrent patients versus cancerous tissues of non‐recurrent patients in the TCGA database. D) The expression levels of these four candidate mRNAs in cancerous lesions of recurrent patients and cancerous tissues of non‐recurrent patients were compared using 34 pairs of fresh frozen tissues obtained after propensity score matching. E) The expression levels of the four candidate mRNAs in peripheral blood of 26 pairs of recurrent patients, non‐recurrent patients, and healthy controls obtained after propensity score matching were compared. F) Western blot analysis results for the four candidate mRNAs in cancer tissues of recurrent and non‐recurrent patients were compared. G) Immunohistochemical detection results of the four candidate mRNAs in cancer tissues of recurrent and non‐recurrent patients were compared. H) The relationship between the expression of the four candidate mRNAs and the clinical pathological characteristics of patients was analyzed. I) An association heat map was generated to analyze the relationship between these four candidate mRNAs and common metastatic genes based on the TCGA database. J) The PPI network of these four candidate mRNAs was constructed using the online STRING database (https://string‐db.org).

In this study, we analyzed transcriptomic data from two publicly available gastric cancer datasets (TCGA and GSE62254) and combined them with mRNA sequencing data from three pairs of LAGC tissues with and without recurrence after radical surgery. We found a significant difference in gene expression between patients with recurrent and non‐recurrent LAGC. Differential gene expression analysis (Wilcoxon rank‐sum test for GSE62254 and EdgeR for TCGA, both with *p* < 0.05 and Bonferroni correction) and correlation analysis (r < 0.5) identified four genes (AGTR1, DNER, EPHA7, and SUSD5) that were differentially expressed (**Figure** **2**A). The volcano plot showed that these four genes were upregulated in recurrent cancer tissues compared to non‐recurrent tissues (Figure 2B).

Further analysis of TCGA data revealed significantly higher expression of these mRNA genes in the recurrent group of LAGC patients than in the non‐recurrent group (P < 0.05) (Figure 2C). A significant correlation was also found between high expression levels of these genes and poorer OS and DFS in these patients (Figure S1B—I, Supporting Informmation). To validate these findings, we established a pilot cohort at FHHMU and analyzed 34 matched tissue samples of recurrent and non‐recurrent primary cancer foci using qRT‐PCR. The results confirmed that the expression levels of these mRNAs were higher in recurrent tissues (P < 0.05) (Figure 2D). Figure 2H results show the relationship between the high and low expression profiles of the four mRNA genes and the clinicopathologic features of the patients in the 34‐case pilot cohort, with high expression of each mRNA strongly correlating with the clinical features of high aggressiveness. Small peripheral blood samples also verified these results (Figure 2E). Western blot analysis showed significantly upregulated expression of these genes in recurrent cancer tissues (Figure 2F; Figure S1J—M, Supporting Informmation). IHC staining further confirmed significantly higher expression levels of these mRNAs in recurrent cancer tissues compared to non‐recurrent tissues (P < 0.05) (Figure 2G).

Protein interaction networks were mapped using the STRING database and visually analyzed with Cytoscape 3.9.1, elucidating the potential roles of these mRNAs in gastric cancer (Figure 2J). Additionally, analysis using the Timer 2.0 database (http://timer.cistrome.org/) revealed positive correlations between these four mRNA genes and metastasis‐related genes MMP2, VEGFC, Vimentin, and N‐cadherin (Figure 2I). Mutational profiling results indicated that the overall tumor mutation burden and the mutation frequency of the gene TTN were higher in the low expression group of AGTR1, SUSD5, and EPHA7, while the opposite trend was observed for DNER (Figure S2, Supporting Informmation).

### 3.2 Validation of the 4‐mRNA Panel in Surgical Resection Specimens of LAGC Patients for Predicting Recurrence

**Figure 3:**
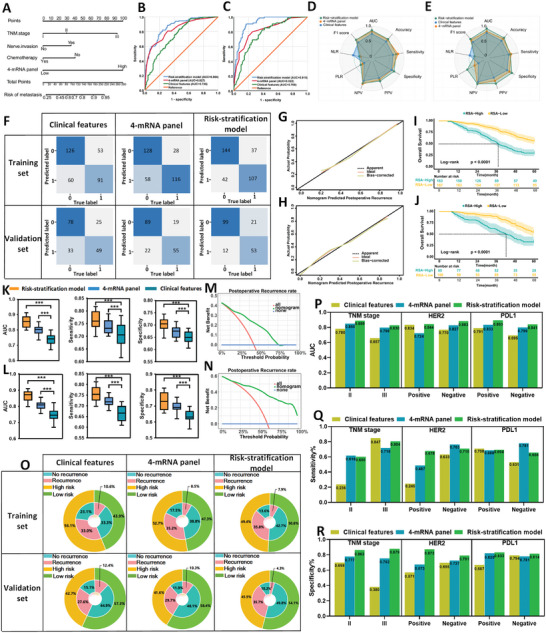
Training and validation of 4‐mRNA prediction based on fresh frozen tissue specimens to identify recurrence in LAGC patients. A) A nomogram for predicting postoperative recurrence in LAGC patients was constructed based on a 4‐mRNA signature combined with clinical features. B) ROC curves of different predictive variables in the training set. C) ROC curves of different predictive variables in the validation set. D) Radar plot comparing evaluation indicators of different prediction models in the training set. E) Radar plot comparing evaluation indicators of different prediction models in the validation set. F) Confusion matrix of different prediction models in the training and validation sets. G) Calibration curve of the RSA model in the training set. H) Calibration curve of the RSA model in the validation set. I) Log‐rank test survival curve of patients in the training set, divided into low‐risk and high‐risk groups according to the critical value obtained from the Youden index of the nomogram. J) Log‐rank test survival curve of patients in the validation set, divided into low‐risk and high‐risk groups. K–L) AUC box plot, sensitivity, and specificity analysis of different prediction models after 1000 bootstrap sessions. M) DCA curve of the RSA model in the training set. N) DCA curve of the RSA model in the validation set. O) Clinical benefit diagram of different prediction models in the training and validation sets. P) Comparison of AUC of different prediction models in a stratified analysis based on different TNM stages and the expression of molecular markers HER2 and PDL1. Q) Comparison of sensitivity of different prediction models in a stratified analysis based on different TNM stages and the expression of molecular markers HER2 and PDL1. R) Comparison of specificity of different prediction models in a stratified analysis based on different TNM stages and the expression of molecular markers HER2 and PDL1.

We first quantified the expression of four mRNA genes using qRT‐PCR in the training cohort. Multivariate analysis revealed that each gene independently influenced the risk of recurrence (all *p* < 0.05, Table S7, Supporting Informmation). Further multivariate analysis identified the 4‐mRNA classifier (OR = 8.466, 95% CI: 4.749‐15.095, *p* < 0.001), TNM staging (OR = 2.136, 95% CI: 1.330‐3.430, *p* = 0.002), nerve invasion (OR = 2.280, 95% CI: 1.209‐4.302, *p* = 0.011), and postoperative chemotherapy (OR = 2.577, 95% CI: 1.176‐5.648, *p* = 0.018) as independent risk factors for recurrence after LAGC (Table S8, Supporting Informmation). Currently, logistic regression is widely used to predict the occurrence of diseases due to its intuitiveness and efficiency.^[^ ^47^, ^48^, ^49^ ^]^ The weight parameters in the model directly reflect the influence of each feature on the classification result. We estimated the probability of recurrence using a logistic regression formula: [(2.136 × 4‐mRNA panel) + (0.759 × TNM staging) + (0.824 × nerve invasion) + (0.947 × postoperative chemotherapy) + (−5.191)]. This model, depicted as a Noetherian pattern, visualized postoperative recurrence prediction in LAGC patients (**Figure** **3**A). Patients were stratified into low and high‐risk categories based on cutoff values determined by the Youden Index.

ROC curve analysis was used to determine the accuracy of individual and combined biomarkers in distinguishing cases of peritoneal recurrence. Although individual mRNA markers were effective, the 4‐mRNA panel showed better diagnostic performance (Figure S3A, Supporting Informmation). Combining the 4‐mRNA panel with clinical variables, we developed a Risk Stratification Assessment (RSA) model that demonstrated excellent predictive ability for recurrence, with an AUC of 0.864 (95% CI: 0.825‐0.902, *p* < 0.001, Figure 3B, D,F upper). Other indicators of comparison between the different models are shown in Table S9 (Supporting Informmation). Notably, the RSA model showed higher AUC values in the training set than the clinical model according to DeLong's test (0.864 vs 0.745; *p* = 0.001). The model's calibration curve further highlighted its predictive accuracy (Figure 3G). A boxplot of AUC after 1000 bootstrap sessions indicated that the RSA model's discriminative ability, sensitivity, and specificity were superior to those of the clinical model and the 4‐mRNA model (Figure 3K).

After calibrating the model using data from the training cohort, the same statistical parameters were applied to the validation cohort. The model maintained consistent statistical parameters and was then applied to an independent external validation cohort of 185 LAGC patients, demonstrating strong predictive ability (AUC = 0.919, 95% CI: 0.881‐0.957, *p* < 0.001) (Figure 3C,E,F lower; Table S9, Supporting Informmation). In addition, the 4‐mRNA panel also showed higher diagnostic performance compared with a single mRNA (Figure S3B, Supporting Informmation). The calibration curve analysis further confirmed the enhanced predictive accuracy (Figure 3H). Similarly, results from 1000 bootstrap iterations for each model showed that the RSA model had the best predictive performance (Figure 3L). Meanwhile, we performed stratified analyses based on different TNM stages, HER2, and PDL1 molecular markers, finding that the RSA model outperformed both the clinical feature model and the 4‐mRNA model in clinical prediction (Figure 3P,Q; Figure S4 and Table S10, Supporting Informmation). However, the sensitivity values of RSA in predicting TNM stage and PDL1 are lower than that of clinical features and mRNA panel (Figure 3Q), which may be a statistical bias caused by a small sample size. We will further expand the sample size and conduct a prospective cohort study in future studies.

The potential of the RSA model in improving the cost‐effectiveness of clinical decision‐making was evaluated using DCA curve results, which indicated good clinical benefits in both the training and validation sets (Figure 3M,N). The double‐layer concentric circle diagram showed that 56.1% of LAGC patients were considered high risk for postoperative recurrence, while 43.9% were classified as low risk. Follow‐up showed that 33.0% (109 of 330 cases) of high‐risk patients and 10.6% (35 of 330 cases) of low‐risk patients experienced recurrence. The RSA model identified more low‐risk patients than the clinical feature model (50.6% vs 43.9%), with only 7.9% of low‐risk patients experiencing recurrence (26/330), compared to 35.8% of high‐risk patients (118/330) (Figure 3O upper). Similar results were obtained in the validation set (Figure 3O lower).

Furthermore, follow‐up of all included LAGC patients for survival showed that the 5 year overall survival (OS) of high‐risk patients was significantly worse than that of low‐risk patients in both the training and validation sets (training set: 30.1% vs 56.9%, P<0.0001; validation set: 32.9% vs 55.0%, *p* < 0.0001) (Figure 3I,J).

### 3.3 Endoscopic Biopsy Specimens to Validate the 4‐mRNA Panel for Predicting Recurrence

**Figure 4:**
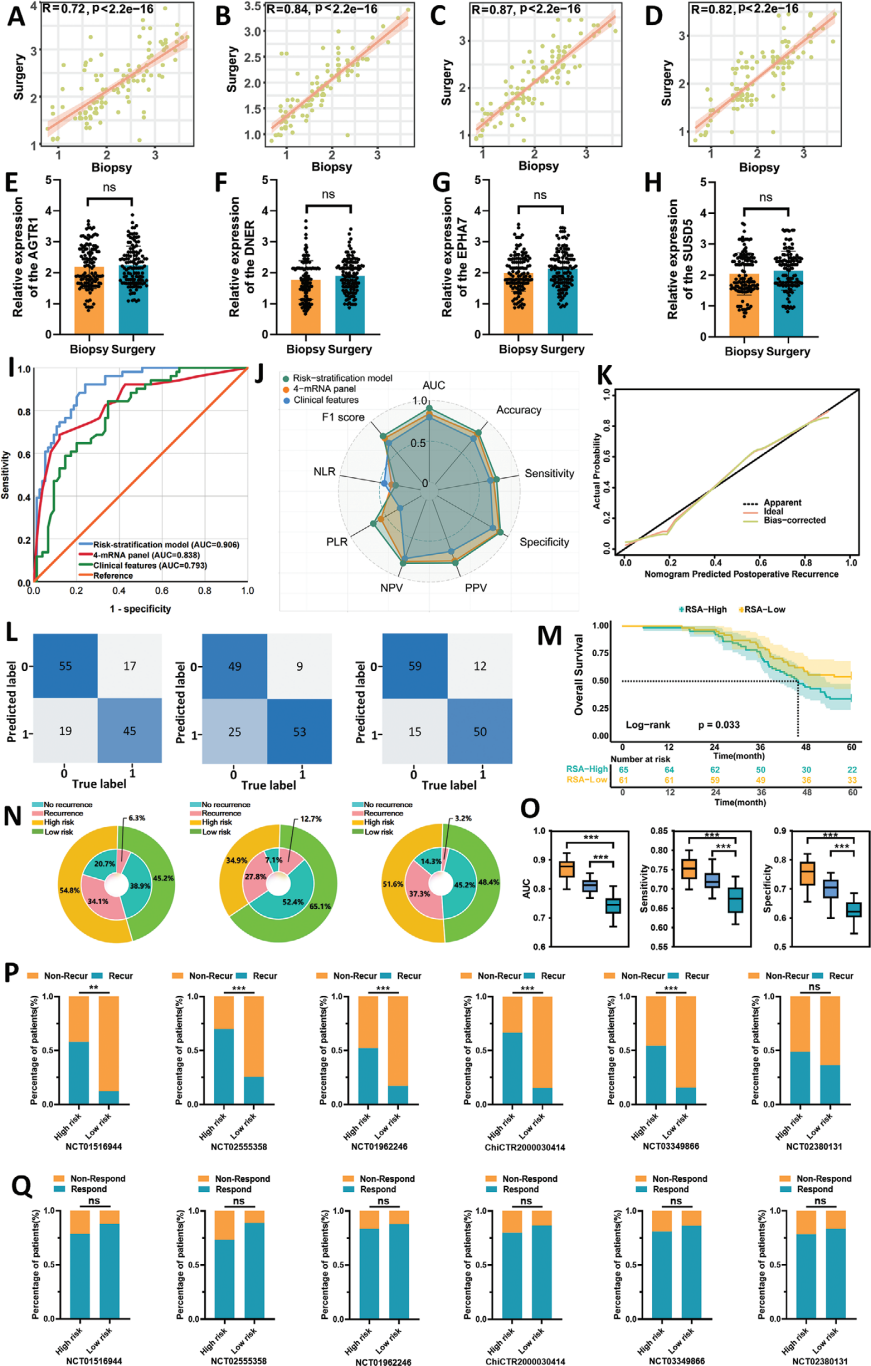
Validation of 4‐mRNA prediction based on endoscopic biopsy specimens to identify recurrence in LAGC patients. A–D) Correlation analysis of the expression of four candidate mRNAs in endoscopic biopsy specimens and paired surgical resection specimens. E–H) Comparison of the expression of four mRNAs in endoscopic biopsy specimens and paired surgical resection specimens. I) ROC curves of different prediction models in endoscopic biopsy specimens. J) Radar plot comparing evaluation indicators of different prediction models in endoscopic biopsy specimens. K) Calibration curve of the RSA model in endoscopic biopsy specimens. L) Confusion matrix between different prediction models. M) Log‐rank test survival curves of patients divided into low‐risk and high‐risk groups according to the cutoff values obtained from the Youden index of the nomogram. N) Clinical benefit diagram of different prediction models in endoscopic biopsy specimens. O) AUC box plot, sensitivity, and specificity analysis of different models after 1000 bootstrap sessions.P) Comparison of the RSA model in predicting postoperative recurrence in LAGC patients in prospective cohorts with different treatment regimens. Q) Comparison of the RSA model in predicting treatment response of LAGC patients in a prospective neoadjuvant treatment cohort with different treatment regimens.

In our study, in addition to the surgical resection specimens in the training and validation cohorts, we obtained 126 matched endoscopic biopsy specimens from six centers, comprising 51 cases with postoperative recurrence and 75 cases without recurrence. The expression profiles of the four genes in biopsy and surgical specimens were highly correlated (**Figure** **4**A–D). No significant differences were found in gene expression between these matched samples (Figure 4E–H). The AUC values and calibration curves confirmed the validity and accuracy of the RSA model (Figure 4I–K; Table S11, Supporting Informmation). In the biopsy cohort, the RSA model demonstrated the highest sensitivity (72.5%) and specificity (89.3%) (Figure 4L). Additionally, the RSA model improved the diagnosis rate of recurrence in the high‐risk group and reduced it in the low‐risk group (Figure 4N), highlighting its potential to enhance clinical decision‐making and minimize unnecessary interventions.

The box plot of AUC values after 1000 bootstrap iterations showed that the RSA model's discrimination ability, sensitivity, and specificity were superior to those of the clinical and 4‐mRNA models (Figure 4O). Similar to the previous cohort follow‐up, a log‐rank test on patients with endoscopic biopsy specimens, categorized into high‐risk and low‐risk groups according to the nomogram, revealed that the 5 year OS was significantly worse in the high‐risk group compared to the low‐risk group (33.8% vs 54.1%, *p* = 0.033) (Figure 4M).

Additionally, we hypothesized that the RSA model could predict the efficacy and recurrence of preoperative neoadjuvant therapy in LAGC patients. Therefore, we included seven prospective neoadjuvant therapy cohorts conducted at FHHMU. The treatment options included neoadjuvant chemotherapy (NCT01516944, NCT02555358), concurrent chemoradiotherapy (NCT01962246), anti‐VEGF‐A targeted therapy (NCT03349866), anti‐HER2 targeted therapy (NCT02380131), and immunotherapy (ChiCTR2000030414). The results showed that, except for the preoperative neoadjuvant anti‐HER2 targeted therapy cohort, the recurrence rate in the high‐risk group was significantly higher than in the low‐risk group in the remaining cohorts (all P < 0.05) (Figure 4P). Furthermore, the proportion of high‐risk patients responding to neoadjuvant therapy was comparable to that of low‐risk patients (all P > 0.05), indicating that the RSA model could not predict the efficacy of preoperative neoadjuvant therapy in LAGC patients (Figure 4Q).

### 3.4 Validation of 4‐mRNA Panels in Peripheral Blood Specimens to Predict Recurrence in LAGC Patients

**Figure 5:**
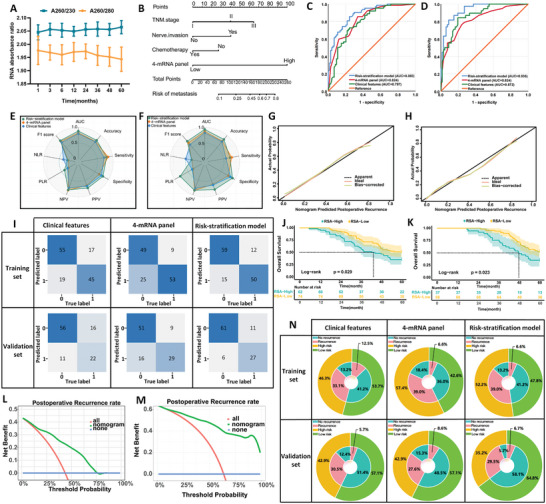
Non‐invasive prediction and identification of LAGC patients with recurrence based on training and validation of 4‐mRNA in peripheral blood specimens. A) Changes in the A260/280 ratio detected at different time points in peripheral blood samples. B) A postoperative recurrence nomogram for LAGC patients constructed based on a 4‐mRNA signature combined with clinical characteristics. C) ROC curves of different prediction models in the training set. D) ROC curves of different prediction models in the validation set. E) Radar chart comparing evaluation indicators of different prediction models in the validation set. F) Confusion matrix of different prediction models in the training set. G) Calibration curve of the RSA model in the training set. H) Calibration curve of the RSA model in the validation set. I) Confusion matrix of different prediction models in both the training and validation sets. J) Log‐rank test survival curve of patients in the training set, divided into low‐risk and high‐risk groups according to the critical value obtained from the Youden index of the nomogram. K) Log‐rank test survival curve of patients in the validation set, divided into low‐risk and high‐risk groups. L) DCA curve of the RSA model in the training set. M) DCA curve of the RSA model in the validation set. N) Clinical benefit diagram of different prediction models in both the training and validation sets.

The main objective of this study was to develop a non‐invasive method based on peripheral blood biopsy to predict postoperative recurrence in patients with LAGC. Initially, 40 patients were randomly selected from the recruited cohort, and quality control analysis was performed on the expression of four mRNAs in their peripheral blood samples. The A260/280 and A260/230 ratios were within the normal range at different time points (**Figure** **5**A). Gel electrophoresis results showed that all four mRNA bands were present, which AGTR1 and DNER had darker bands due to lower mRNA detection values (Figure S5, Supporting Informmation), indicating that the mRNA in the peripheral blood was not degraded and suitable for qRT‐PCR analysis.

Multivariate logistic regression revealed that high expression of the four mRNAs was a risk factor for postoperative recurrence in LAGC patients (all P < 0.05, Table S7, Supporting Informmation). A nomogram for predicting postoperative recurrence was constructed based on multivariate logistic regression analysis (Figure 5B), and the AUC for the 4‐mRNA biomarker group was 0.824 (95% CI: 0.755–0.893, *p* < 0.001; Figure 5C), indicating high predictive accuracy. The risk probability formula for postoperative recurrence was calculated using the logistic regression coefficients: [(3.250 × 4‐mRNA panel) + (1.908 × TNM stage) + (1.798 × nerve invasion) + (2.512 × postoperative chemotherapy) + (−9.625)]. Notably, the liquid biopsy‐based RSA model outperformed the clinical and 4‐mRNA models in predicting recurrence (Figures 5E,I upper; Table S12, Supporting Informmation), and the calibration curve analysis confirmed its excellent performance (Figure 5G). Comparative clinical benefit analysis revealed that the RSA model increased the recurrence detection rate in the high‐risk group from 33.1% to 39.0% and reduced the recurrence detection rate in the low‐risk group from 12.5% to 6.6% (Figure 5L,N upper). Patients stratified into low‐risk and high‐risk groups based on the Youden index exhibited significantly different 5 year OS rates (35.5% vs 51.4%, *p* = 0.029) (Figure 5J).

The RSA model's predictive ability was further validated in an independent cohort, yielding an AUC of 0.935 (95% CI: 0.891‐0.979, *p* < 0.001) (Figure 5D). Radar chart and confusion matrix analyses confirmed the RSA model's superiority over the four‐mRNA panel and clinical feature models in predicting recurrence (Figure 5F,I lower; Table S12, Supporting Informmation). Calibration curve analysis also confirmed the improved predictive performance of the RSA model (Figure 5H). These results demonstrate that the tissue‐based four‐mRNA group can be successfully applied to liquid biopsy detection using peripheral blood specimens, and the RSA model, combining serum four‐mRNA data and clinical features, effectively predicts postoperative recurrence in LAGC patients. Decision curve analysis and double‐layer concentric circle diagrams further confirmed the RSA model's potential in improving clinical decision‐making and optimizing patient management (Figure 5M,N lower). Meanwhile, follow‐up revealed that the 5 year OS in the high‐risk group of the RSA model was significantly lower than in the low‐risk group (35.1% vs 52.9%, *p* = 0.023) (Figure 5K).

### 3.5 4‐mRNA Panel for Dynamic Monitoring of Postoperative Recurrence in LAGC Patients

**Figure 6:**
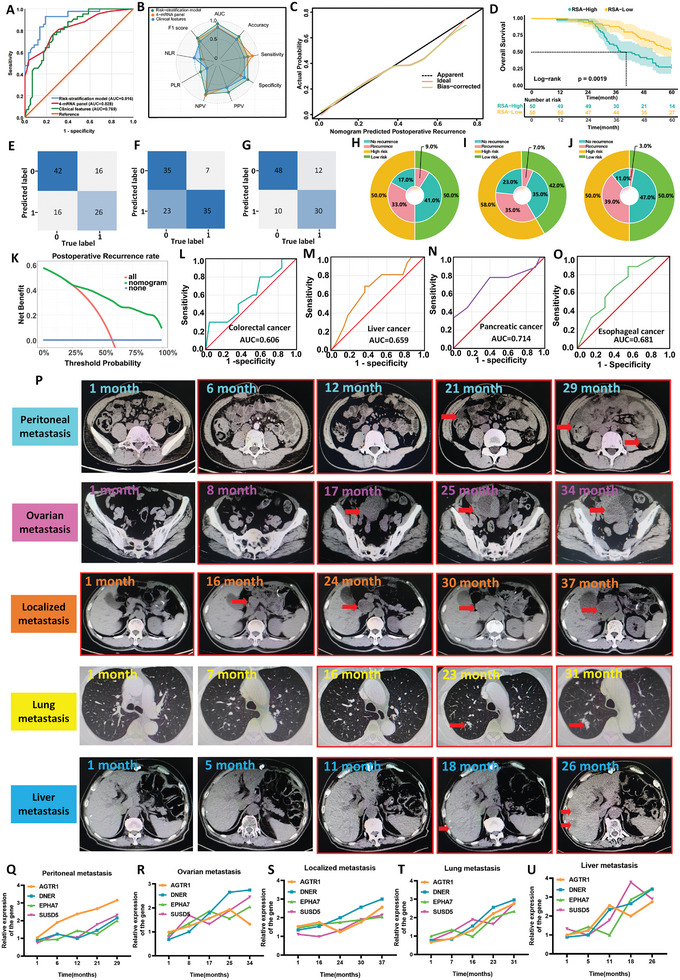
Validation of 4‐mRNA recognition based on peripheral blood samples to predict tumor marker‐negative patients and longitudinal dynamic prediction to identify different types of recurrence. A) ROC curves of different prediction models in the validation set of tumor marker‐negative patients. B) Radar chart comparing evaluation indicators of different prediction models in the validation set of tumor marker‐negative patients. C) Calibration curve of the RSA model in the validation set of tumor marker‐negative patients. D) Log‐rank test survival curves of tumor marker‐negative patients, divided into low‐risk and high‐risk groups according to the critical value obtained from the Youden index of the nomogram. E–G) Confusion matrices of the RSA model constructed using clinical characteristics, 4‐mRNA, and their combination to predict recurrence in tumor marker‐negative patients. H–J) Clinical benefit diagrams of the RSA model constructed using clinical characteristics, 4‐mRNA, and their combination to predict recurrence in tumor marker‐negative patients. K) DCA curve of the RSA model in the tumor marker‐negative patient set. L) ROC curve of the 4‐mRNA signature for predicting postoperative recurrence in colorectal cancer patients. M) ROC curve of the 4‐mRNA signature for predicting postoperative recurrence in hepatocellular carcinoma patients. N) ROC curve of the 4‐mRNA signature for predicting postoperative recurrence in pancreatic cancer patients. O) ROC curve of the 4‐mRNA signature for predicting postoperative recurrence in patients with esophageal cancer. P) The time when recurrence was detected by traditional CT. Q–U) The time of recurrence predicted by the longitudinal dynamic changes of the four mRNAs for patients with the common metastatic forms.

In current clinical practice, peripheral blood tumor markers such as CA19‐9, CA72‐4, and CEA are commonly used to monitor postoperative recurrence in patients with LAGC.^[^ ^50^, ^51^, ^52^ ^]^ To evaluate the ability of the RSA model to identify patients with negative tumor markers who are likely to experience postoperative recurrence, we analyzed peripheral blood samples from patients with negative tumor marker results in the combined training and validation cohorts, which included 58 patients without postoperative recurrence and 42 patients with recurrence. We validated the nomogram developed in the training cohort, and the results demonstrated that the RSA model (AUC = 0.916, 95% CI: 0.862–0.970) was significantly superior to the clinical feature model (AUC = 0.769; 95% CI: 0.727–0.893) and the 4‐mRNA panel model (AUC = 0.828, 95% CI: 0.746–0.911) in predicting postoperative recurrence (**Figure** **6**A,B; Table S13, Supporting Informmation). The confusion matrix (Figure 6E–G) and calibration curve (Figure 6C) analysis confirmed the RSA model's higher accuracy in predicting postoperative recurrence compared to other models. We analyzed the ability of different models to identify recurrence between different risk groups and found that the RSA model significantly outperformed clinical features and the 4‐mRNA panel in detecting postoperative recurrence (Figure 6H–K). Furthermore, among patients with negative tumor markers, the five‐year OS in the high‐risk group was significantly lower than in the low‐risk group (28.0% vs 54.0%, *p* = 0.0019) (Figure 6D).

Furthermore, we evaluated the diagnostic performance of the 4‐mRNA panel in predicting postoperative recurrence using serum samples from patients with other gastrointestinal malignancies, including colorectal cancer (*n* = 35), liver cancer (*n* = 38), pancreatic adenocarcinoma (*n* = 29), and esophageal cancer (*n* = 29). Compared to its performance in predicting postoperative recurrence in LAGC patients, the RSA model exhibited inferior predictive ability for postoperative recurrence in colorectal cancer (AUC = 0.606), liver cancer (AUC = 0.659), pancreatic adenocarcinoma (AUC = 0.714), and esophageal cancer (AUC = 0.681) (Figure 6L–O). The DeLong test confirmed the RSA model's higher specificity for LAGC than for other gastrointestinal cancers (P < 0.001).

In addition, to further verify the predictive performance of the RSA model in the longitudinal dynamic changes of LAGC patients, we selected five patients who experienced recurrence after radical surgery, including peritoneal metastasis, ovarian metastasis, local tumor bed metastasis, lung metastasis, and liver metastasis. These metastatic forms are common in LAGC patients after surgery. We collected peripheral blood from these five patients during postoperative follow‐up and analyzed and compared the difference between the time of recurrence predicted by the longitudinal dynamic changes of the 4‐mRNA panel and the time of recurrence detected by traditional CT imaging. The red frame indicates an increase in the four‐mRNA levels, suggesting a high‐risk trend for recurrence. The red arrow indicates the time when recurrence was detected by traditional CT. As shown in the results of Figure 6P, compared with traditional CT detection, the RSA model constructed by combining the four‐mRNA panel with clinical characteristics can achieve early warning of recurrence through longitudinal dynamic monitoring, particularly for the common metastatic forms encountered in clinical practice (Figure 6Q–U).

### 3.6 Recurrence mRNA Panel Genes Promote GC Proliferation and Metastasis In Vitro and In Vivo

**Figure 7:**
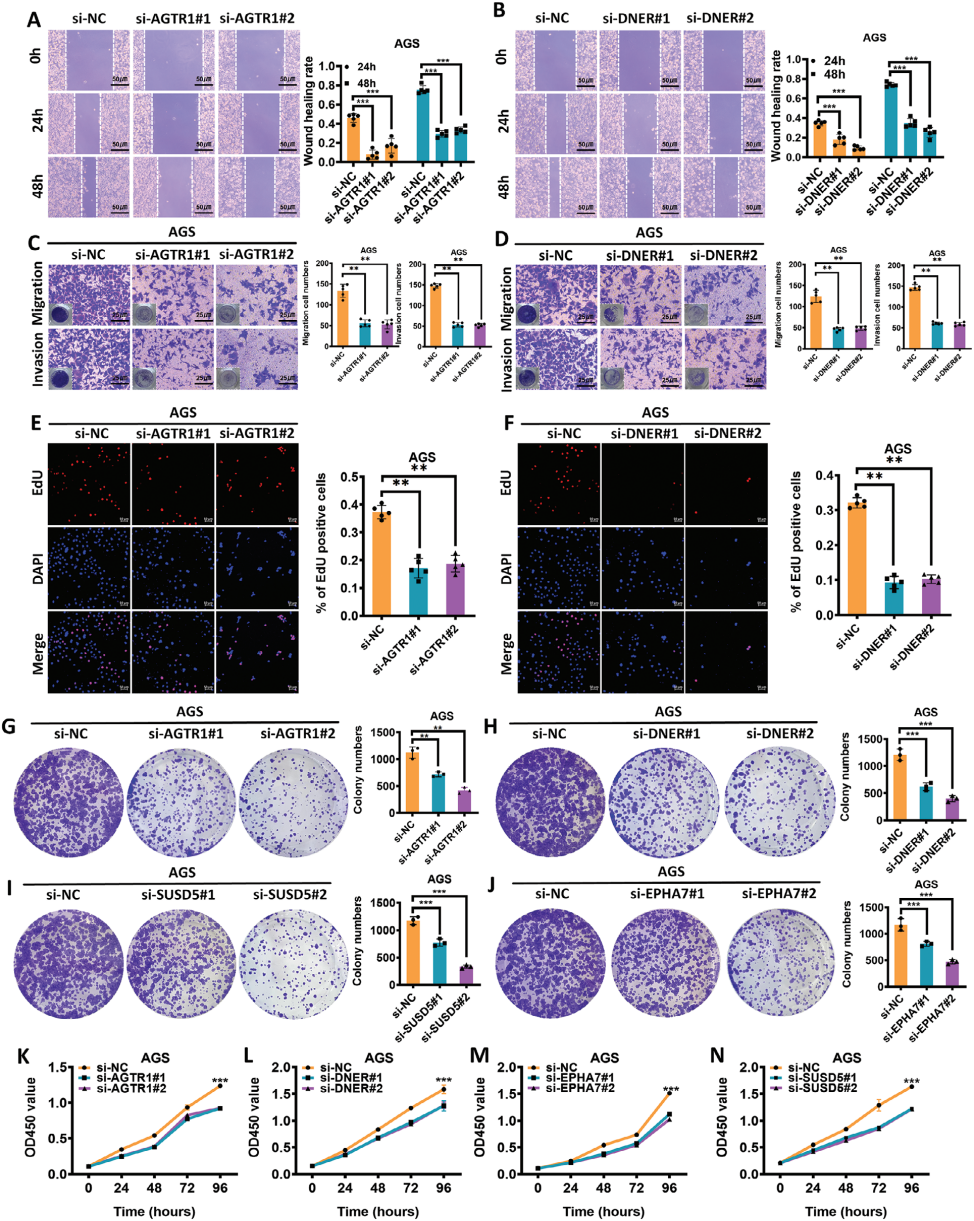
Four recurrence‐related mRNA genes promote GC cell proliferation, migration and invasion in vitro. A,B) Scratch assay to evaluate the migration ability of GC cells after knockdown of AGTR1 and DNER, respectively. C,D) Transwell assay to assess the invasion and metastasis abilities of GC cells after knockdown of AGTR1 and DNER, respectively. E,F) EdU assay to determine the proliferation ability of GC cells after knockdown of AGTR1 and DNER, respectively. G–J) Colony formation assay to measure the proliferation ability of GC cells after knockdown of AGTR1, DNER, EPHA7, and SUSD5. K–N) CCK‐8 assay to detect the proliferation ability of GC cells after knockdown of AGTR1, DNER, EPHA7, and SUSD5. ^*^ *P* < 0.05, ^**^ *P* < 0.01, ^***^ *P* < 0.001.

Considering the potential clinical value of the 4‐mRNA panel in predicting postoperative recurrence in patients with LAGC, we investigated the biological effects of the four mRNA genes (AGTR1, DNER, EPHA7, and SUSD5) comprising this panel. Specific siRNA sequences targeting these genes (sequences shown in Table S14, Supporting Informmation) were employed. Scratch healing and transwell assays demonstrated that silencing AGTR1, DNER, EPHA7, and SUSD5 significantly impaired the migratory and invasive capacities of AGS and HGC‐27 cells (**Figure** **7**A–D;Figure S6A–D and S7, Supporting Informmation). Moreover, EdU assays revealed diminished DNA replication activity in AGS and HGC‐27 cells transfected with these siRNAs compared to control cells (Figure 7E,F; Figure S6E,F and S8A—D, Supporting Informmation). Colony formation and CCK‐8 assays further indicated that proliferation (Figure 7K–N; Figure S8I—L, Supporting Informmation) and clonogenic potential (Figure 7G–J; Figure S8E—H, Supporting Informmation) were markedly reduced upon silencing these genes.

**Figure 8:**
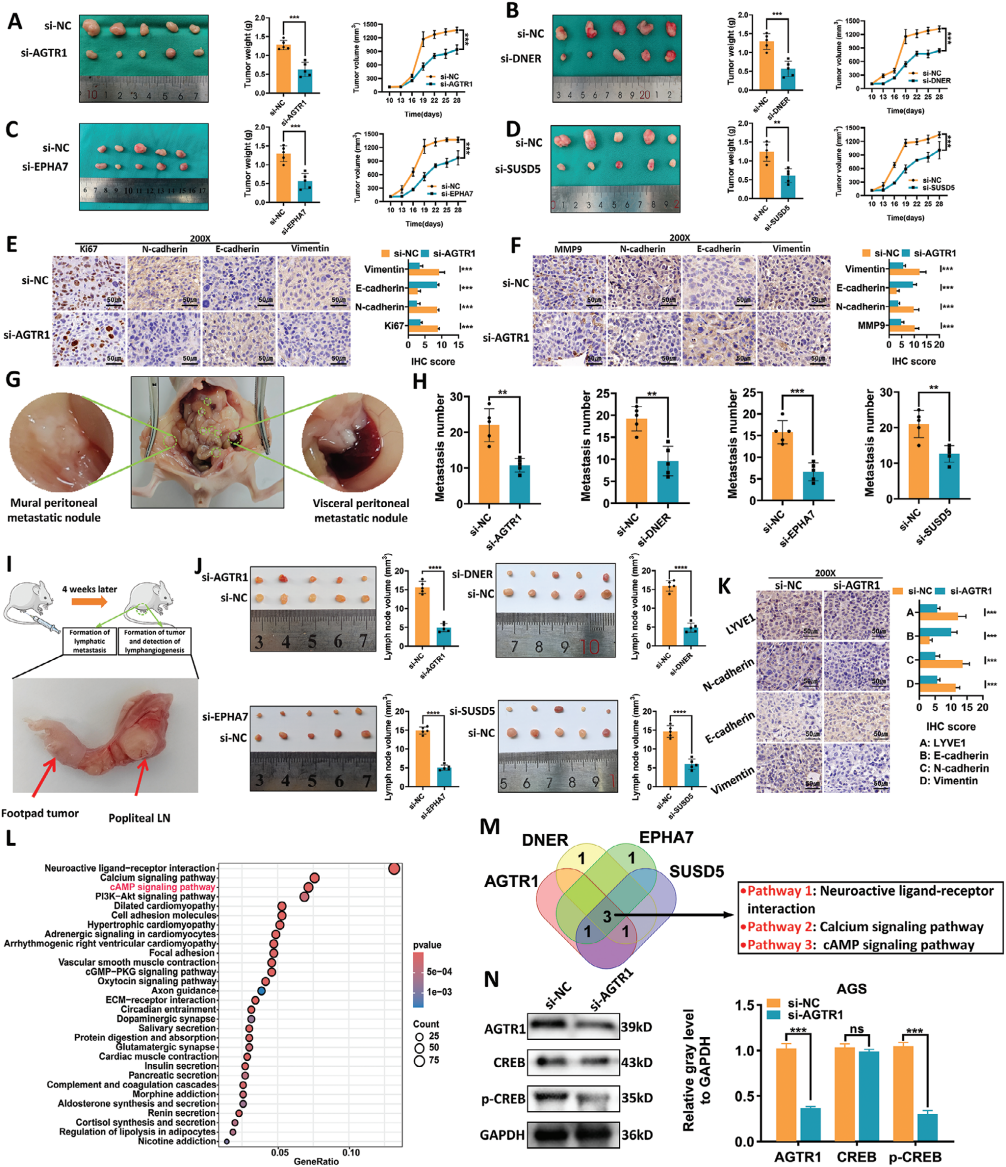
Four recurrence‐related mRNA genes promote GC cell xenograft tumor growth and metastasis in vivo. A–D) Morphological images showing reduced subcutaneous xenograft tumor formation in mice injected with AGS cells knocked down for AGTR1, DNER, EPHA7, and SUSD5, along with tumor volume growth curves and final tumor weights. E) Representative IHC images of subcutaneous xenograft tumors after knockdown of AGTR1 (left), and quantification of IHC staining data for Ki67, N‐cadherin, E‐cadherin, and Vimentin in each group of mice (right). F) Representative IHC images of peritoneal metastasis tumors after intraperitoneal injection of AGS cells knocked down for AGTR1 (left), and quantification of IHC staining data for MMP9, N‐cadherin, E‐cadherin, and Vimentin in each group of mice (right). G) Representative images of peritoneal metastasis tumors in the abdominal cavity of mice injected with AGS cells knocked down for AGTR1. H) Measurement and quantification of the number of peritoneal metastatic tumors in mice after intraperitoneal injection of AGS cells with knockdown of AGTR1, DNER, EPHA7, and SUSD5. I) Representative images of popliteal lymph node metastasis in mice after footpad injection of AGS cells with knockdown of AGTR1. J) Morphological images showing reduced popliteal lymph node formation and final lymph node volume measurement in mice after injection of AGS cells with knockdown of AGTR1, DNER, EPHA7, and SUSD5. K) Representative IHC images of popliteal lymph node metastasis after footpad injection of AGS cells with knockdown of AGTR1 (left), and quantification of IHC staining data for LYVE1, N‐cadherin, E‐cadherin, and Vimentin in each group of mice (right). L) KEGG bubble diagram of the AGTR1 gene. M) Venn diagram of KEGG pathway enrichment for four recurrence‐related mRNA genes. N) Determination of the protein expression status of CREB and p‐CREB in AGS cells after AGTR1 knockdown (left) and further quantification (right).^*^ *P* < 0.05, ^**^ *P* < 0.01, ^***^ *P* < 0.001.

We performed in vivo knockdown experiments targeting AGTR1, DNER, EPHA7, and SUSD5 in BALB/c nude mice to investigate the effects of these recurrence mRNA panel genes on GC tumor growth and metastasis. Subcutaneous tumor growth and weight were diminished in the siRNA groups compared to controls (**Figure** **8**A–D). IHC analysis of primary subcutaneous xenograft tumors revealed decreased expression of the proliferation marker Ki‐67 and the mesenchymal markers N‐cadherin and vimentin, coupled with enhanced expression of the epithelial marker E‐cadherin upon knockdown of these genes (Figure 8E; Figure S9, Supporting Informmation). Peritoneal metastases were significantly reduced in the siRNA groups relative to controls (Figure 8G,H). Consistent with the subcutaneous xenograft tumors model, IHC staining demonstrated that silencing these genes reduced the expression of metastatic (MMP9) and mesenchymal (N‐cadherin, vimentin) markers while increasing the expression of epithelial (E‐cadherin) markers (Figure 8F; Figure S10, Supporting Informmation). Furthermore, analysis of primary footpad tumors and popliteal lymph nodes revealed diminished lymph node metastatic burden in the siRNA groups (Figure 8I,J). Remarkably, IHC analysis of popliteal lymph nodes showed reduced expression of the lymphangiogenic marker LYVE1, mesenchymal markers (N‐cadherin, vimentin), and increased epithelial marker (E‐cadherin) expression following knockdown of AGTR1, DNER, EPHA7, and SUSD5 (Figure 8K; Figure S11, Supporting Informmation). Collectively, these in vitro and in vivo findings indicate that the recurrence of mRNA panel genes enhances migration, invasion, epithelial‐mesenchymal transition, lymphangiogenesis, and metastasis of GC.

Further, Kyoto Encyclopedia of Genes and Genomes (KEGG) pathway enrichment analysis revealed that all four recurrence mRNA panel genes were associated with neuroactive ligand‐receptor interaction, calcium signaling, and cAMP signaling pathways (Figure 8L; Figure S12B,D,F, Supporting Informmation). Notably, numerous literature searches on PubMed (https://pubmed.ncbi.nlm.nih.gov/) indicated that the cAMP signaling pathway is closely linked to gastric cancer recurrence(Figure 8M; Figures S12A, Supporting Informmation). Consistent with this finding, western blotting demonstrated decreased phosphorylation of the key cAMP pathway effector CREB upon knockdown of the four recurrence mRNA panel genes, suggesting their potential pro‐oncogenic effects are mediated through cAMP signaling (Figure 8N; Figure S12C,E,G, Supporting Informmation).

## 4 Discussion

The findings of this study highlight the development and validation of a transcriptomics‐based liquid biopsy panel for the early detection of recurrence in patients with LAGC. The study identified a panel of four mRNAs (AGTR1, DNER, EPHA7, and SUSD5) significantly upregulated in recurrent gastric cancer tissues compared to non‐recurrent tissues. This panel was validated across multiple cohorts, demonstrating high predictive accuracy for recurrence. The RSA model, integrating the 4‐mRNA panel with clinical variables, showed superior performance in predicting recurrence compared to traditional clinical models. The model achieved an AUC of 0.864 in the training set and 0.919 in the validation cohort, underscoring its potential as a non‐invasive, highly sensitive, and specific tool for monitoring LAGC patients post‐surgery.

Previous studies have explored the potential of liquid biopsy approaches for gastric cancer management, primarily focusing on circulating tumor cells (CTCs) and cell‐free DNA (cfDNA).^[^ ^26^, ^27^, ^28^, ^29^, ^30^, ^31^, ^32^ ^]^ However, these traditional liquid biopsy components have faced challenges in sensitivity and specificity. For instance, a meta‐analysis by Yao et al. reported a pooled sensitivity of only 32.6% for CTCs in detecting gastric cancer recurrence.^[^ ^53^ ^]^ Similarly, a study by Creemers et al. found that ctDNA had limited diagnostic accuracy for early‐stage gastric cancer.^[^ ^54^ ^]^ In contrast, the present study leverages the dynamic and comprehensive nature of transcriptomic biomarkers, offering a promising alternative for early recurrence detection. Moreover, while previous studies have proposed individual mRNA or miRNA biomarkers for gastric cancer prognosis,^[^ ^38^, ^39^, ^40^, ^41^ ^]^ this study presents a multi‐biomarker panel approach, which may enhance diagnostic performance.

Notably, the RSA model's predictive performance in this study surpasses that of several previously reported models. For instance, a study by Kim et al. developed a nomogram for predicting recurrence in gastric cancer based on clinical and pathological factors, achieving an AUC of 0.82 in the validation cohort.^[^ ^55^ ^]^ In contrast, the RSA model in the present study achieved AUCs of 0.919 and 0.935 in the surgical and liquid biopsy validation cohorts, respectively. Similarly, a study by Lee et al. reported a nomogram based on clinical factors and a four‐protein signature for predicting recurrence in gastric cancer, with an AUC of 0.86 in the validation set.^[^ ^56^ ^]^ The superior performance of the RSA model in the current study highlights the potential advantages of integrating transcriptomic biomarkers with clinical features for improved predictive accuracy.

Interestingly, the study also provides insights into the potential molecular mechanisms underlying the pro‐oncogenic effects of the four recurrence mRNA panel genes. At present, numerous literatures have reported that these four genes are closely related to the malignant progression of gastric cancer.^[^ ^57^, ^58^, ^59^, ^60^, ^61^ ^]^ In this study, functional experiments revealed that silencing these genes impaired the migratory, invasive, and proliferative capabilities of gastric cancer cells in vitro, while in vivo xenograft models demonstrated reduced tumor growth, metastasis, and lymphangiogenesis upon gene knockdown. Notably, pathway enrichment analysis implicated these genes in neuroactive ligand‐receptor interaction, calcium signaling, and cAMP signaling pathways, with the latter being closely linked to gastric cancer recurrence based on literature evidence. Indeed, western blotting confirmed decreased phosphorylation of the key cAMP effector CREB upon gene silencing, suggesting that the pro‐oncogenic effects of these genes may be mediated through the cAMP signaling pathway. These findings not only elucidate the functional roles of the recurrence mRNA panel genes but also provide potential therapeutic targets for preventing or treating gastric cancer recurrence.

While the study presents a promising approach for early recurrence detection in LAGC, it is essential to acknowledge certain limitations and potential areas for future improvement. First, the study primarily focused on evaluating the predictive performance of the 4‐mRNA panel and RSA model but did not delve into the specific clinical implications or decision‐making algorithms based on risk stratification. Future studies could explore how this approach can be integrated into clinical practice and guide personalized treatment strategies for LAGC patients. Second, although the study demonstrated the specificity of the RSA model for LAGC recurrence prediction, it would be valuable to further evaluate its performance in other cancer types to assess its potential broader applicability. Furthermore, considering the complexity of transcriptomic regulation in cancer, future research should delve into the interplay between the identified mRNA biomarkers and other regulatory molecules, such as microRNAs (miRNAs) or long non‐coding RNAs (lncRNAs). Understanding these interactions could offer a more comprehensive perspective on the molecular mechanisms underlying gastric cancer recurrence. This avenue of exploration could lead to the identification of additional biomarkers or therapeutic targets, enhancing the predictive power of the RSA model. In conclusion, acknowledging these limitations and exploring these avenues for improvement will not only strengthen the current study's findings but also contribute to advancing the field of cancer prognosis and treatment.

## 5 Conclusion

In conclusion, this study presents a groundbreaking approach for the early detection of recurrence in locally advanced gastric cancer through a transcriptomics‐based liquid biopsy panel. The development of the RSA model, integrating a 4‐mRNA panel with clinical features, demonstrated superior predictive performance compared to traditional clinical models or single‐biomarker approaches. The functional characterization of the recurrence mRNA panel genes provided insights into their roles in promoting gastric cancer progression and metastasis, potentially through the cAMP signaling pathway. Despite certain limitations, this study represents a significant advancement in the field of liquid biopsy and personalized medicine for gastric cancer management. The non‐invasive, sensitive, and specific nature of this approach holds promise for enabling continuous monitoring of LAGC patients, facilitating early interventions and personalized treatment strategies, ultimately improving patient outcomes and reducing the burden associated with traditional diagnostic methods.

## Conflict of Interest

The authors declare no conflict of interest.

## Author Contributions

Q.Z. and L.M. performed conception and design. Q.Z. performed administrative support. P.D., H.W., J.W., T.L., J.H., X.N., H.G., Y.T., P.Y., J.Y., R.G., MD, L.Z., N.M., X.L., Z.G., L.M, Q.Z. performed provision of study materials or patients. H.W., J.W., T.L. performed collection and assembly of data. P.D., H.W., J.W performed data analysis and interpretation. P.D., L.M., H.W., J.W. wrote the final manuscript and final approval of manuscript.

## Supporting information



Supporting Information

Supporting Information

## Data Availability

The data that support the findings of this study are available from the corresponding author upon reasonable request.
