# Supplementary material for: Transcriptomics‐Based Liquid Biopsy for Early Detection of Recurrence in Locally Advanced Gastric Cancer
Source: Adv Sci (Weinh). 2024 Nov 18;11(47):2406276. doi: 10.1002/advs.202406276 (PMC11653671; doi:10.1002/advs.202406276)
Supplement: Supplementary file 1 — Supporting Information [file ADVS-11-2406276-s001.docx]

**SI Figures and Tables**


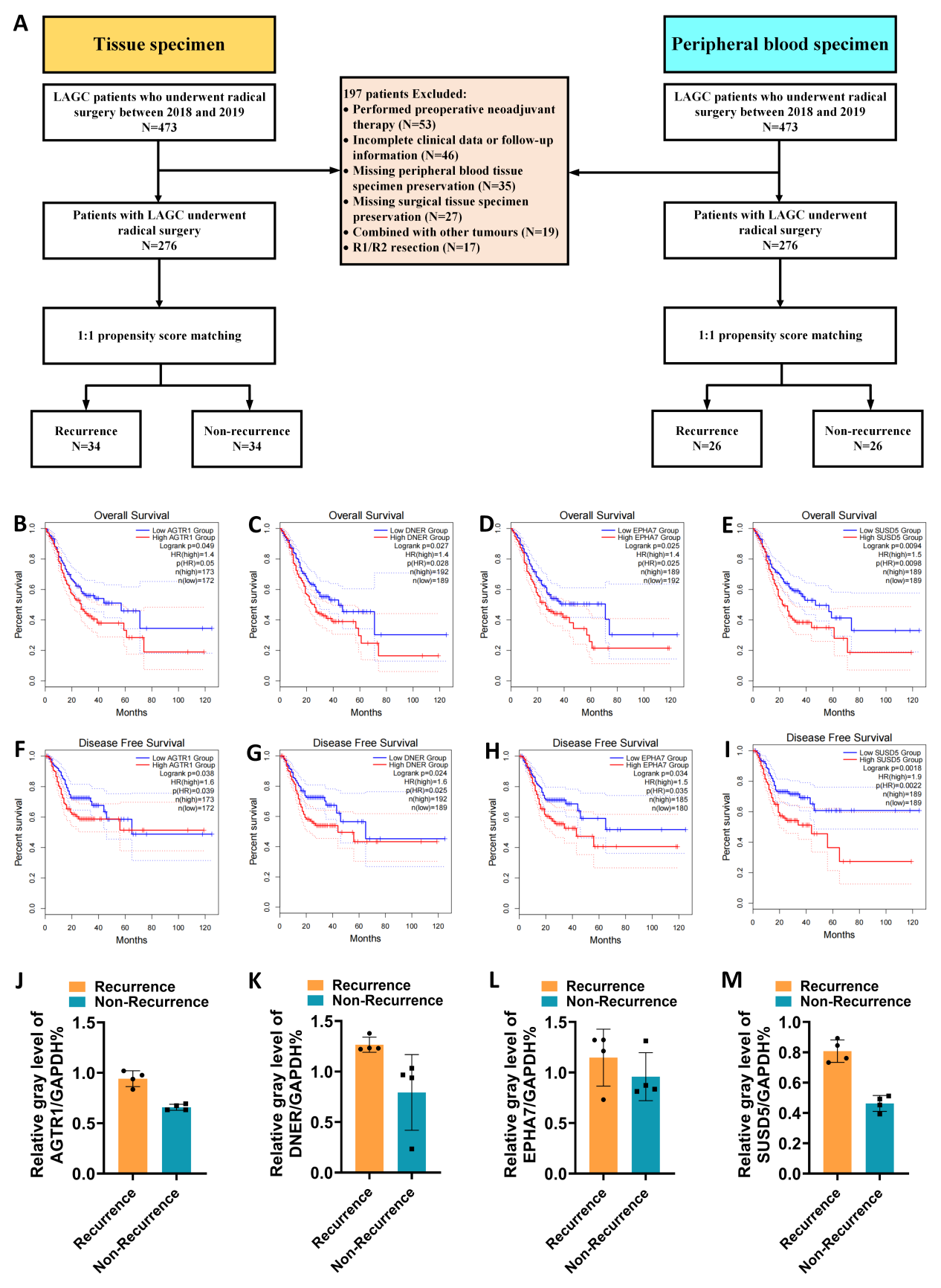


**Figure S1. Cohort validation, prognostic analysis, and protein quantitative expression of the four candidate mRNA genes**. (A) Flowchart of propensity score matching of surgical tissue specimens and peripheral blood specimens in the pilot cohort. (B-E) Overall survival analysis curves of the four candidate mRNA genes in the TCGA database. (F-I) Disease-free survival analysis curves of the four candidate mRNA genes in the TCGA database. (J-M) Quantitative analysis of Western blot detection results of the four candidate mRNAs in cancer tissues of recurrent and non-recurrent patients.


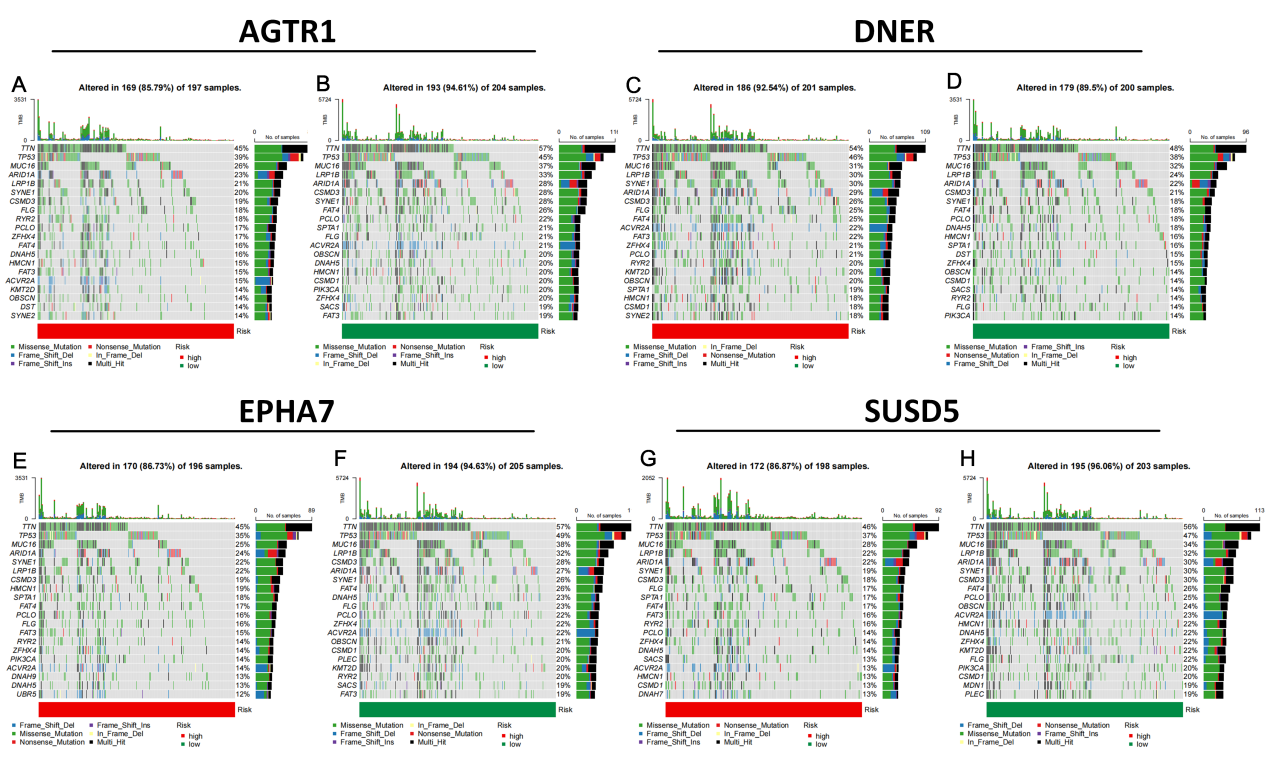


**Figure S2. Mutation Map Analysis of Four Candidate mRNA Genes.** Mutation maps are shown for high and low expression groups of each candidate gene: (A-B) AGTR1, (C-D) DNER, (E-F) EPHA7, and (G-H) SUSD5.


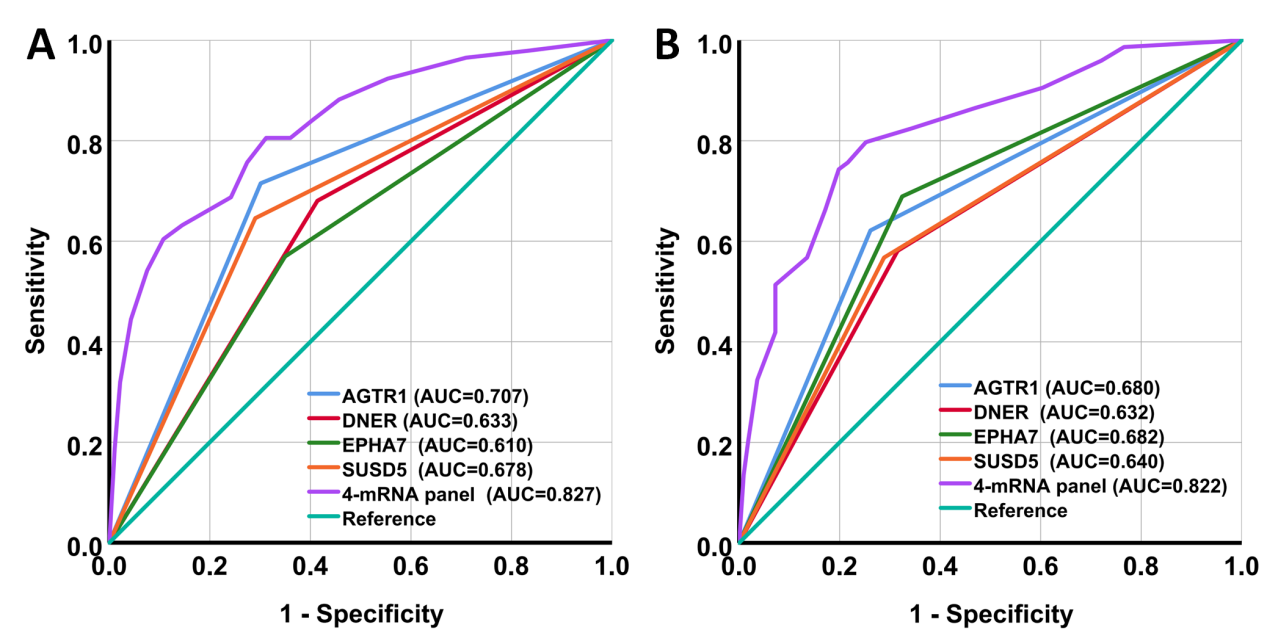


**Figure S3. ROC curves of 4 mRNAs and 4-mRNA panel.** (A) ROC curves of different predictive variables in the training set. (B) ROC curves of different predictive variables in the validation set.


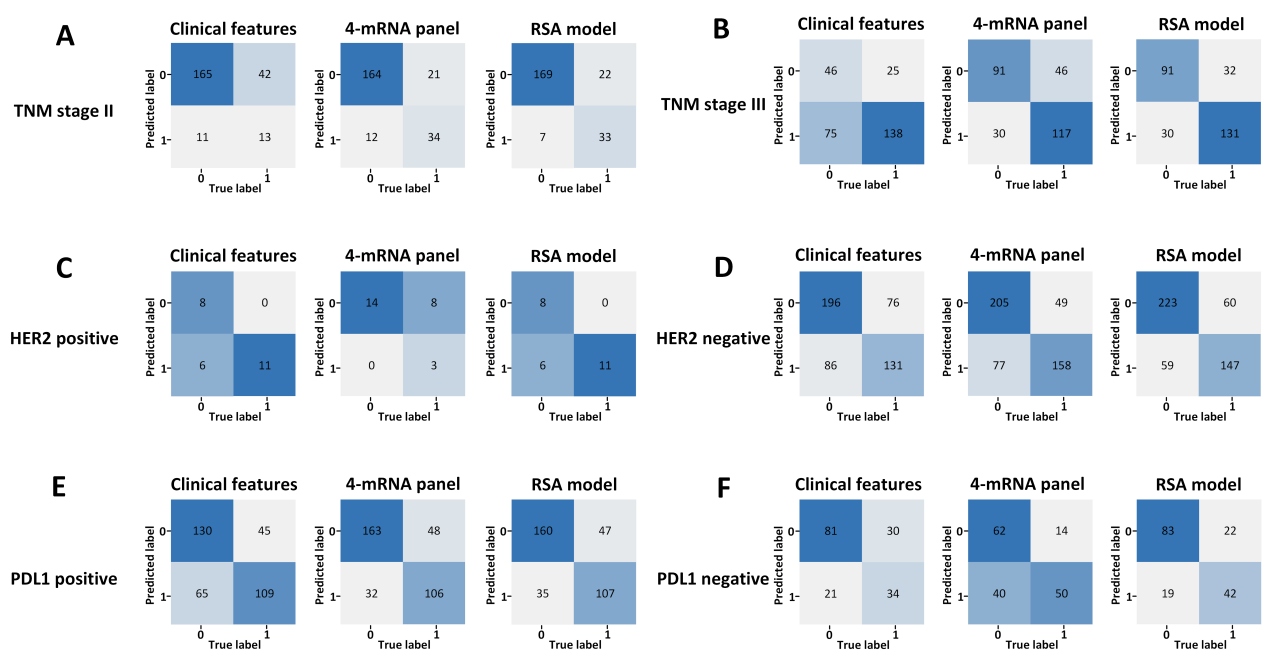


**Figure S4. Confusion matrix diagram of stratified analysis of subgroups based on different TNM stages and different expressions of molecular markers HER2 and PDL1.** (A) Confusion matrix diagram of different prediction models for predicting postoperative recurrence in patients with TNM stage II. (B) Confusion matrix diagram of different prediction models for predicting postoperative recurrence in patients with TNM stage III. (C) Confusion matrix diagram of different prediction models for predicting postoperative recurrence in patients with positive expression of molecular marker HER2. (D) Confusion matrix diagram of different prediction models for predicting postoperative recurrence in patients with negative expression of molecular marker HER2. (E) Confusion matrix diagram of different prediction models for predicting postoperative recurrence in patients with positive expression of molecular marker PDL1. (F) Confusion matrix diagram of different prediction models for predicting postoperative recurrence in patients with negative expression of molecular marker PDL1.


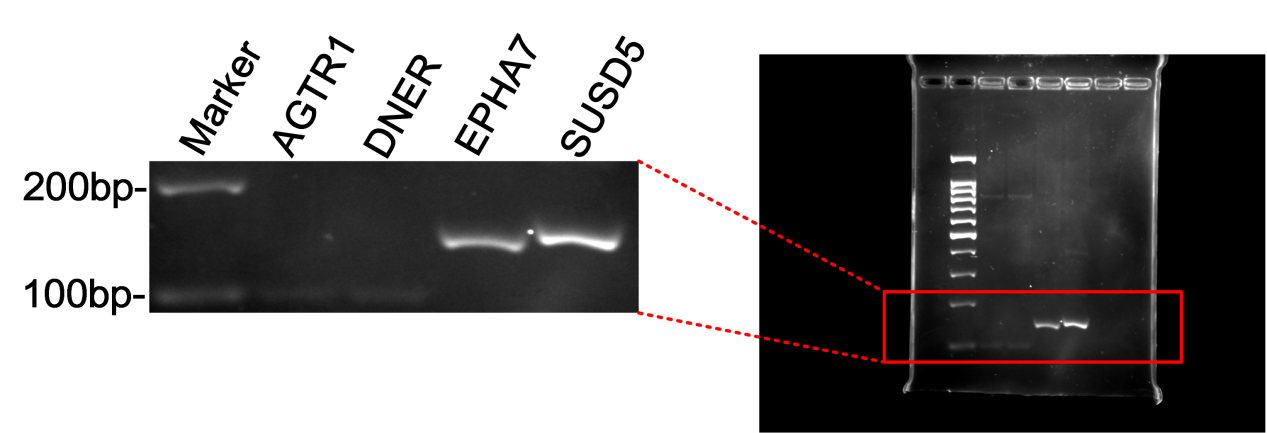


**Figure S5. Gel electrophoresis of four candidate mRNA genes in peripheral blood specimens.**


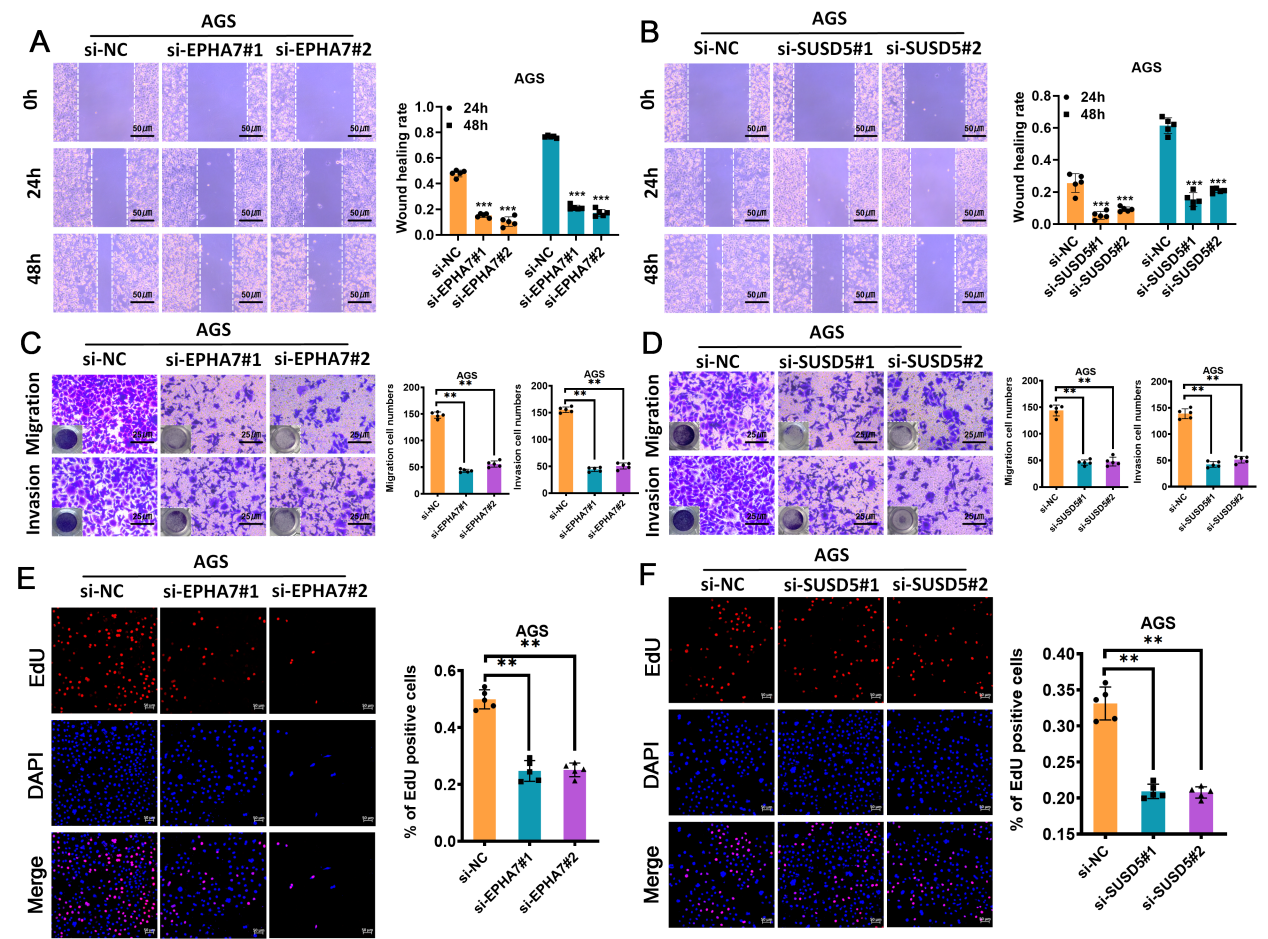


**Figure S6. Candidate mRNA genes promote GC cell proliferation, migration and invasion in vitro.** (A-B) Scratch assay of GC cells under knockdown of EPHA7 and SUSD5, respectively, to detect the migration ability. (C-D) Transwell assay of GC cells under knockdown of EPHA7 and SUSD5, respectively, to detect invasion and metastasis ability. (E-F) EdU assay of GC cells under knockdown of EPHA7 and SUSD5, respectively, to detect proliferation ability. **P*<0.05, ***P*<0.01, ****P*<0.001.


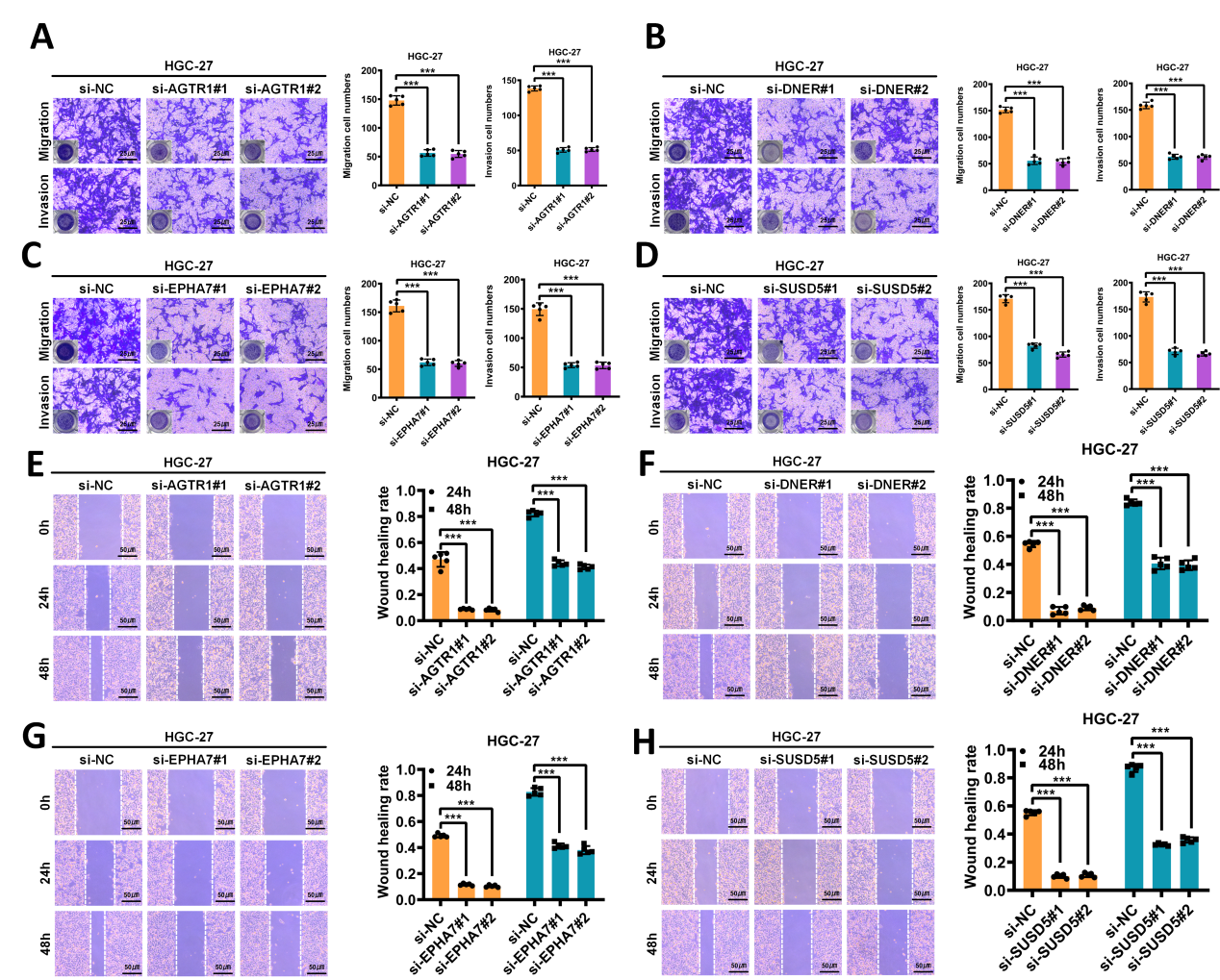


**Figure S7. Candidate mRNA genes promote HGC-27 cells migration and invasion in vitro.** (A-D) Transwell assay of HGC-27 cells under knockdown of AGTR1, DNER, EPHA7 and SUSD5, respectively, to detect invasion and metastasis ability. (E-H) Scratch assay of HGC-27 cells under knockdown of AGTR1, DNER, EPHA7 and SUSD5, respectively, to detect the migration ability. **P*<0.05, ***P*<0.01, ****P*<0.001.


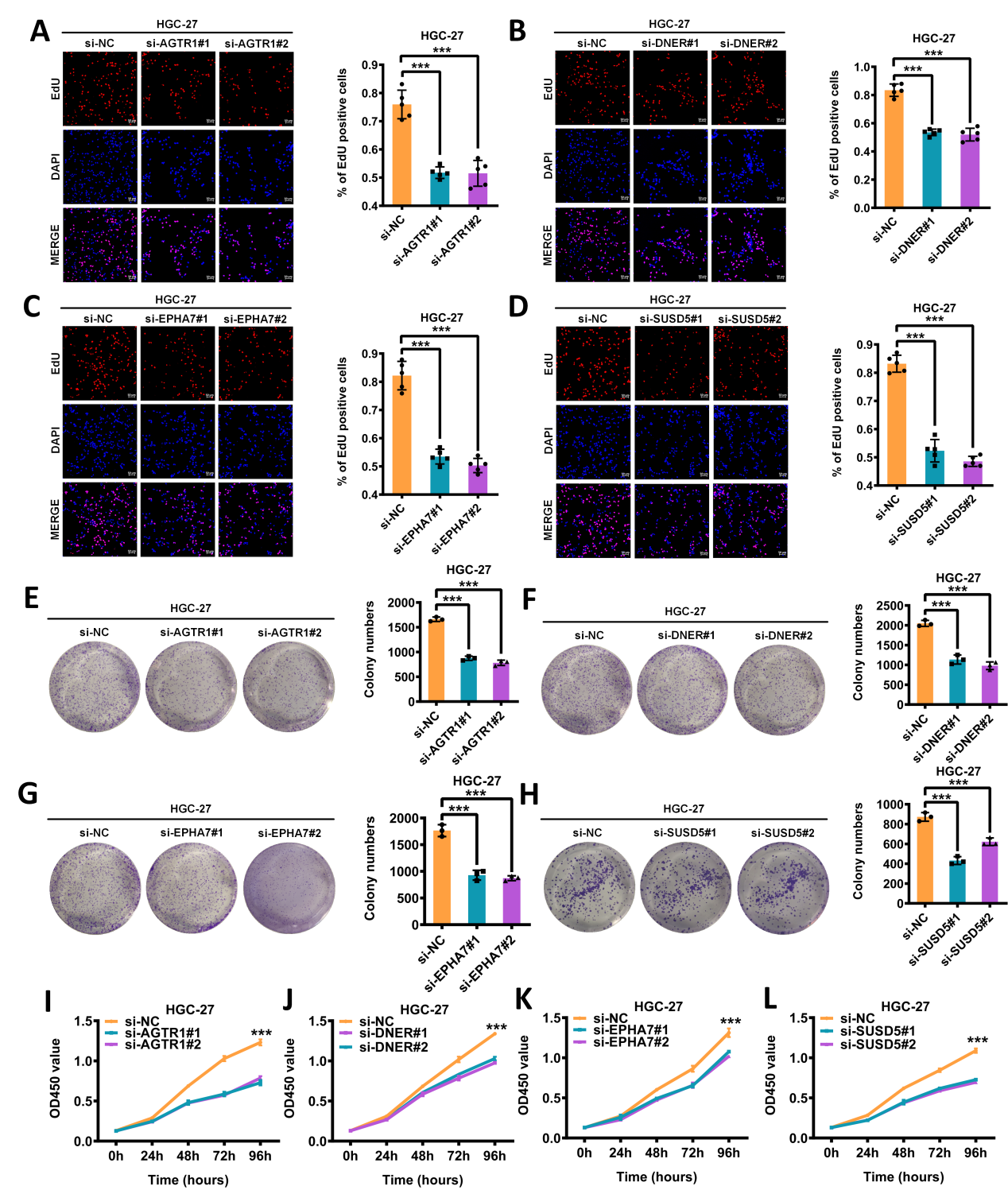


**Figure S8. Four recurrence-related mRNA genes promote HGC-27 cells proliferation in vitro.** (A-D) EdU assay of HGC-27 cells under knockdown of AGTR1, DNER, EPHA7 and SUSD5, respectively, to detect proliferation ability. (E-H) Colony formation assay to measure the proliferation ability of HGC-27 cells after knockdown of AGTR1, DNER, EPHA7, and SUSD5. (I-L) CCK-8 assay to detect the proliferation ability of HGC-27 cells after knockdown of AGTR1, DNER, EPHA7, and SUSD5. *P<0.05, **P<0.01, ***P<0.001.


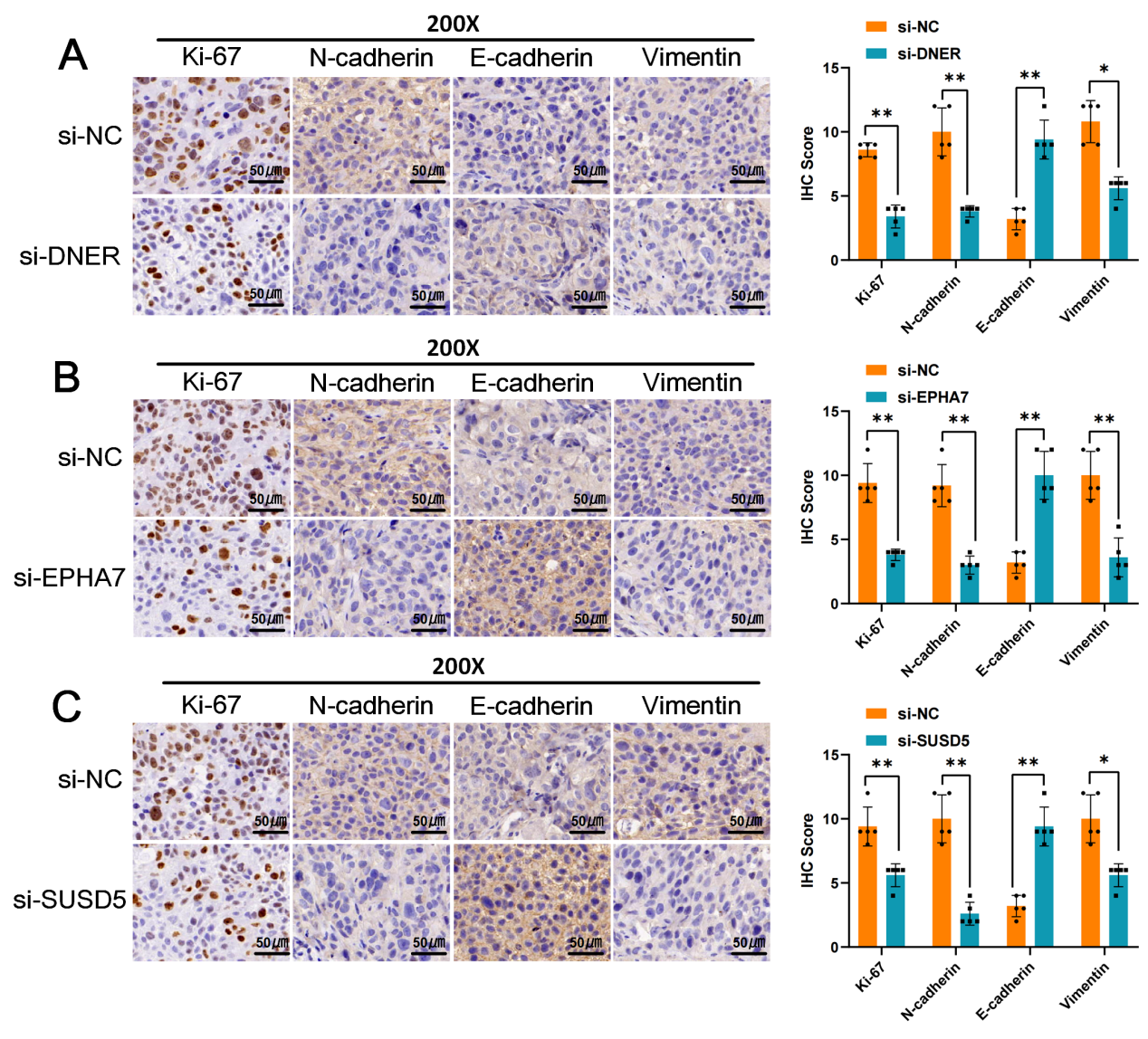


**Figure S9. Candidate mRNA genes promote GC cell xenograft tumor growth and metastasis in vivo.** (A) Representative IHC images of injected knockdown DNER subcutaneous xenograft tumors (left), and quantification of IHC staining data for Ki67, N-cadherin, E-cadherin, and Vimentin for each group of mice (right). (B) Representative IHC images of subcutaneous xenograft tumors injected with knockdown EPHA7 (left), and quantification of IHC staining data for Ki67, N-cadherin, E-cadherin, and Vimentin for each group of mice (right). (C) Representative IHC images of subcutaneous xenograft tumors injected with knockdown SUSD5 (left), and quantification of IHC staining data for Ki67, N-cadherin, E-cadherin, and Vimentin for each group of mice (right). **P*<0.05, ***P*<0.01, ****P*<0.001.


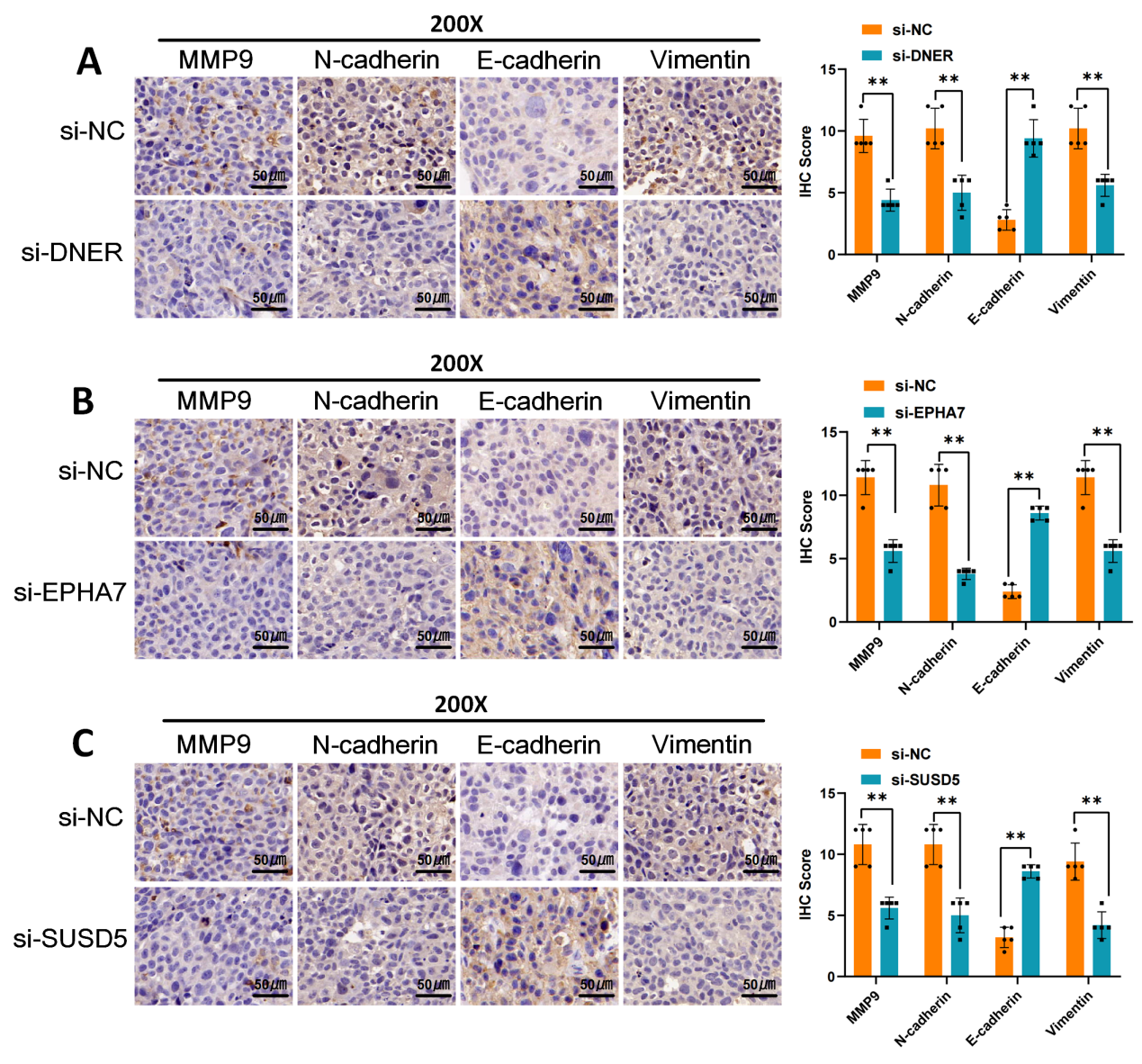


**Figure S10.** **Candidate mRNA genes promote intraperitoneal peritoneal metastatic tumor growth and metastasis of GC cells in vivo.** (A) Representative IHC images of peritoneal metastatic tumors after intraperitoneal injection of AGS cells knocking down DNER (left), and quantification of IHC staining data for MMP9, N-cadherin, E-cadherin, and Vimentin in each group of mice (right). (B) Representative IHC images of peritoneal metastatic tumors after intraperitoneal injection of AGS cells knocked down for EPHA7 (left), and quantification of IHC staining data for MMP9, N-cadherin, E-cadherin, and Vimentin for each group of mice (right). (C) Representative IHC images of peritoneal metastatic tumors after intraperitoneal injection of AGS cells knocking down SUSD5 (left), and quantification of IHC staining data of MMP9, N-cadherin, E-cadherin, and Vimentin for each group of mice (right). **P*<0.05, ***P*<0.01, ****P*<0.001.


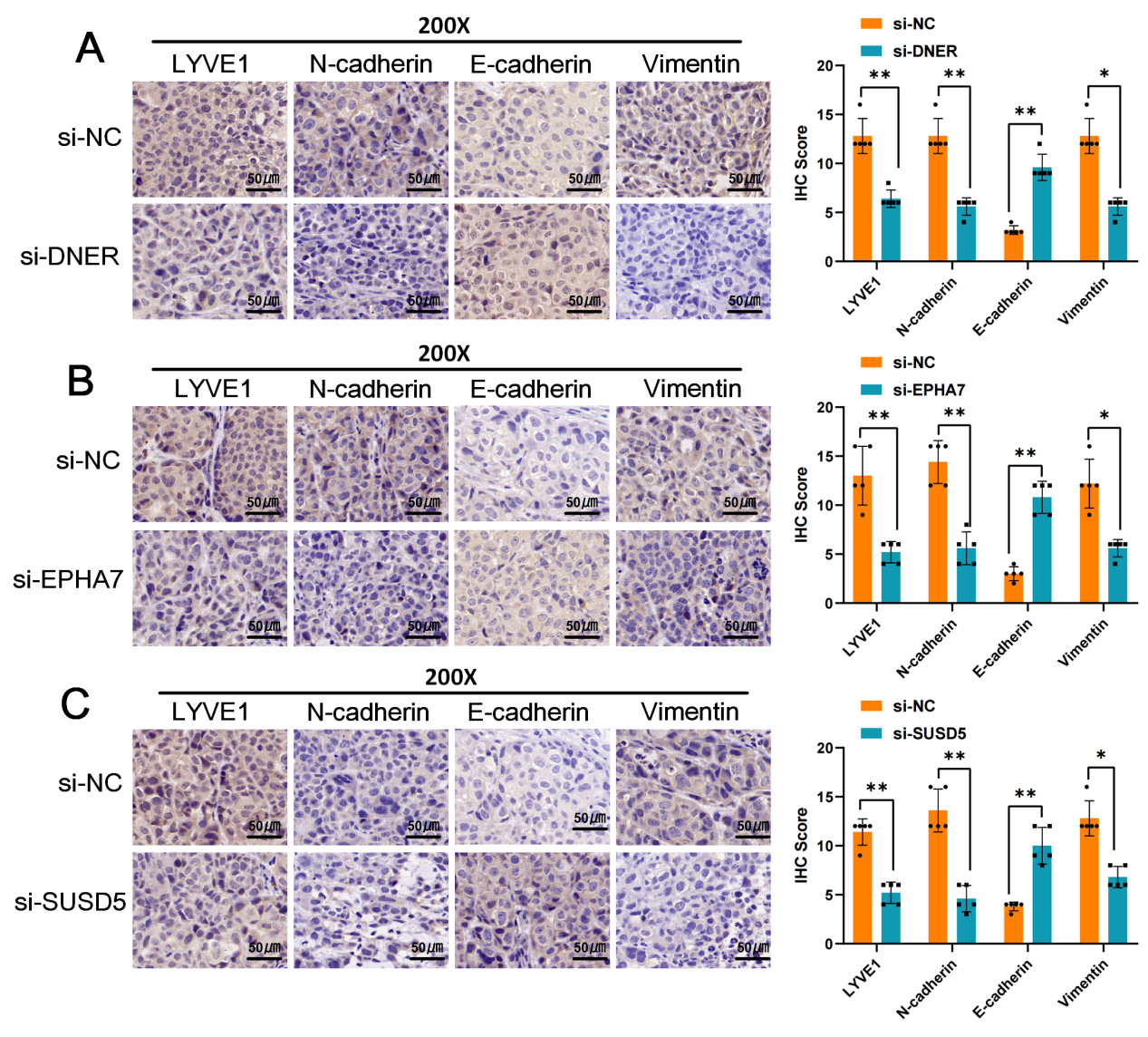


**Figure S11. Candidate mRNA genes promote popliteal lymph node metastasis of GC cells in vivo.** (A) Representative IHC images of popliteal lymph node metastasis in AGS cells after footpad injection knockdown of DNER (left), and quantification of IHC staining data of LYVE1, N-cadherin, E-cadherin, and Vimentin in each group of mice (right). (B) Representative IHC images of popliteal lymph node metastasis after footpad injection of AGS cells after knockdown of EPHA7 (left), and quantification of IHC staining data of LYVE1, N-cadherin, E-cadherin, and Vimentin for each group of mice (right). (C) Representative IHC images of popliteal lymph node metastasis after footpad injection of AGS cells after knockdown of SUSD5 (left), and quantification of IHC staining data of LYVE1, N-cadherin, E-cadherin, and Vimentin for each group of mice (right). **P*<0.05, ***P*<0.01, ****P*<0.001.


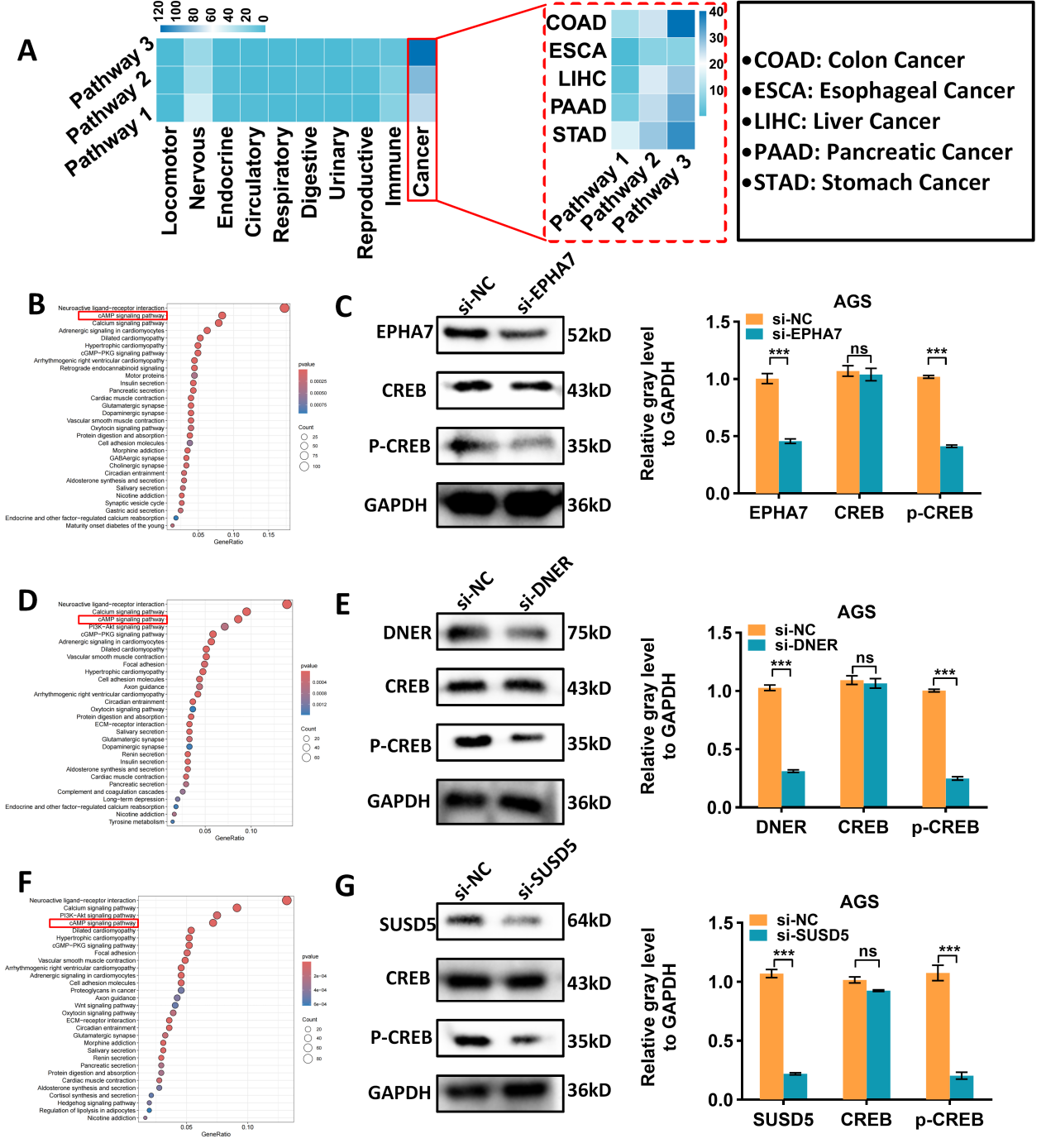


**Figure S12. Pathway enrichment and validation of candidate mRNA genes.** (A) Heatmap trend of literature publication of the potential role pathways of four candidate mRNA genes in gastric cancer development. (B) KEGG bubble map of EPHA7 gene. (C) Protein expression status of CREB, p-CREB in AGS cells after EPHA7 knockdown was determined (left) and further quantified (right). (D) KEGG bubble map of DNER gene. (E) Protein expression status of CREB, p-CREB in AGS cells after DNER knockdown was determined (left) and further quantified (right). (F) KEGG bubble map of SUSD5 gene. (G) Protein expression status of CREB, p-CREB in AGS cells after SUSD5 knockdown was determined (left) and further quantified (right). **P*<0.05, ***P*<0.01, ****P*<0.001.

**Table S1. Analysis of clinicopathological characteristics of patients with surgical specimens before and after propensity score matching [n (%)]**

| **Clinical characteristic** | **Before propensity score matching** | |  | **After propensity score matching** | |
| --- | --- | --- | --- | --- | --- |
|  | **Non-recurrence**  **(n=234)** | **Recurrence**  **(n=42)** |  | **Recurrence**  **(n=34)** | **Non-recurrence**  **(n=34)** |
| **Gender** |  |  |  |  |  |
| Male | 149 (63.7) | 26 (61.9) |  | 17 (50.0) | 19 (55.9) |
| Female | 85 (36.3) | 16 (38.1) |  | 17 (50.0) | 15 (44.1) |
| **Age(years)** |  |  |  |  |  |
| ≤65 | 128 (54.7) | 32 (76.2) |  | 23 (67.6) | 25 (73.5) |
| ＞65 | 106 (45.3) | 10 (23.8) |  | 11 (32.4) | 8 (26.5) |
| **T stage** |  |  |  |  |  |
| T2/T3 | 61 (26.1) | 6 (14.3) |  | 6 (17.6) | 5 (14.7) |
| T4 | 173 (73.9) | 36 (85.7) |  | 28 (82.4) | 29 (85.3) |
| **N stage** |  |  |  |  |  |
| N0 | 46 (19.7) | 8 (19.0) |  | 7 (20.6) | 6 (17.6) |
| N+ | 188 (80.3) | 34 (81.0) |  | 27 (79.4) | 28 (82.4) |
| **Primary site** |  |  |  |  |  |
| Up 1/3 | 86 (36.8) | 14 (33.3) |  | 11 (32.4) | 12 (35.3) |
| Middle 1/3 | 34 (14.5) | 7 (16.7) |  | 6 (17.6) | 4 (11.8) |
| Lower 1/3 | 114 (48.7) | 21 (50.0) |  | 17 (50.0) | 18 (52.9) |
| **Tumor size(cm)** |  |  |  |  |  |
| ≤5 | 66 (28.2) | 11 (26.2) |  | 7 (20.6) | 6 (17.6) |
| ＞5 | 168 (71.8) | 31 (73.8) |  | 27 (79.4) | 28 (82.4) |
| **Histology** |  |  |  |  |  |
| None/Low | 198 (84.6) | 35 (83.3) |  | 31 (91.2) | 32 (94.1) |
| High/Median | 36 (15.4) | 7 (16.7) |  | 3 (8.8) | 2 (5.9) |
| **Lauren** |  |  |  |  |  |
| Diffuse/Mix type | 188 (80.3) | 33 (78.6) |  | 26 (76.5) | 27 (20.6) |
| Intestinal type | 46 (19.7) | 9 (21.4) |  | 8 (23.5) | 7 (79.4) |
| **Vascular invasion** |  |  |  |  |  |
| Yes | 127 (54.3) | 32 (76.2) |  | 25 (73.5) | 26 (76.5) |
| No | 107 (45.7) | 10 (23.8) |  | 9 (26.5) | 8 (23.5) |
| **Nerve invasion** |  |  |  |  |  |
| Yes | 100 (42.7) | 26 (61.9) |  | 23 (67.6) | 24 (70.6) |
| No | 134 (57.3) | 16 (38.1) |  | 11 (32.4) | 10 (29.4) |

**Table S2. Analysis of clinicopathological characteristics of patients with peripheral blood samples before and after propensity score matching**

| **Clinical characteristic** | **Before propensity score matching** | | |  | **After propensity score matching** | | |
| --- | --- | --- | --- | --- | --- | --- | --- |
|  | **Non-recurrence**  **(n=234)** | | **Recurrence**  **(n=42)** |  | **Non-recurrence**  **(n=26)** | | **Recurrence**  **(n=26)** |
| **Gender** |  |  | |  |  |  | |
| Male | 149 (63.7) | 26 (61.9) | |  | 16 (61.5) | 17 (65.4) | |
| Female | 85 (36.3) | 16 (38.1) | |  | 10 (38.5) | 9 (34.6) | |
| **Age(years)** |  |  | |  |  |  | |
| ≤65 | 128 (54.7) | 32 (76.2) | |  | 19 (73.1) | 19 (73.1) | |
| ＞65 | 106 (45.3) | 10 (23.8) | |  | 7 (26.9) | 7 (26.9) | |
| **T stage** |  |  | |  |  |  | |
| T2/T3 | 61 (26.1) | 6 (14.3) | |  | 3 (11.5) | 2 (7.7) | |
| T4 | 173 (73.9) | 36 (85.7) | |  | 23 (88.5) | 24 (92.3) | |
| **N stage** |  |  | |  |  |  | |
| N0 | 46 (19.7) | 8 (19.0) | |  | 5 (19.2) | 6 (23.1) | |
| N+ | 188 (80.3) | 34 (81.0) | |  | 21 (80.8) | 20 (76.9) | |
| **Primary site** |  |  | |  |  |  | |
| Up 1/3 | 86 (36.8) | 14 (33.3) | |  | 9 (34.6) | 8 (30.8) | |
| Middle 1/3 | 34 (14.5) | 7 (16.7) | |  | 4 (15.4) | 5 (19.2) | |
| Lower 1/3 | 114 (48.7) | 21 (50.0) | |  | 13 (50.0) | 13 (50.0) | |
| **Tumor size(cm)** |  |  | |  |  |  | |
| ≤5 | 66 (28.2) | 11 (26.2) | |  | 4 (15.4) | 5 (19.2) | |
| ＞5 | 168 (71.8) | 31 (73.8) | |  | 22 (84.6) | 21 (80.8) | |
| **Histology** |  |  | |  |  |  | |
| None/Low | 198 (84.6) | 35 (83.3) | |  | 25 (96.2) | 25 (96.2) | |
| High/Median | 36 (15.4) | 7 (16.7) | |  | 1 (3.8) | 1 (3.8) | |
| **Lauren** |  |  | |  |  |  | |
| Diffuse/Mix type | 188 (80.3) | 33 (78.6) | |  | 19 (73.1) | 20 (76.9) | |
| Intestinal type | 46 (19.7) | 9 (21.4) | |  | 7 (26.9) | 6 (23.8) | |
| **Vascular invasion** |  |  | |  |  |  | |
| Yes | 127 (54.3) | 32 (76.2) | |  | 18 (69.2) | 19 (73.1) | |
| No | 107 (45.7) | 10 (23.8) | |  | 8 (30.8) | 7 (26.9) | |
| **Nerve invasion** |  |  | |  |  |  | |
| Yes | 100 (42.7) | 26 (61.9) | |  | 15 (57.7) | 16 (61.5) | |
| No | 134 (57.3) | 16 (38.1) | |  | 11 (42.3) | 10 (38.5) | |

**Table S3. Clinical characteristics of training set and validation set of surgical resection specimen cohort[n(%)]**

| **Clinical characteristic** | **Training cohort**  **(N=330)** | **Validation cohort**  **(N=185)** | **P value** |
| --- | --- | --- | --- |
| **Gender** |  |  | 0.724 |
| Male | 205 (62.1) | 112 (60.5) |  |
| Female | 125 (37.9) | 73 (39.5) |  |
| **Age(years)** |  |  | 0.889 |
| ≤65 | 168 (50.9) | 93 (50.3) |  |
| ＞65 | 162 (49.1) | 92 (49.7) |  |
| **T stage** |  |  | 0.181 |
| T2/T3 | 151 (45.8) | 96 (51.9) |  |
| T4 | 179 (54.2) | 89 (48.1) |  |
| **N stage** |  |  | 0.267 |
| N0 | 99 (30.0) | 47 (25.4) |  |
| N+ | 231 (70.0) | 138 (74.6) |  |
| **Primary site** |  |  | 0.965 |
| Up 1/3 | 107 (32.4) | 61 (33.0) |  |
| Middle 1/3 | 96 (29.1) | 55 (29.7) |  |
| Lower 1/3 | 127 (38.5) | 69 (37.3) |  |
| **Tumor size(cm)** |  |  | 0.810 |
| ≤5 | 132 (40.0) | 72 (38.9) |  |
| ＞5 | 198 (60.0) | 113 (61.1) |  |
| **Histology** |  |  | 0.771 |
| None/Low | 297 (90.0) | 165 (89.2) |  |
| High/Median | 33 (10.0) | 20 (10.8) |  |
| **Lauren** |  |  | 0.486 |
| Diffuse/Mix type | 210 (63.6) | 112 (60.5) |  |
| Intestinal type | 120 (36.4) | 73 (39.5) |  |
| **Vascular invasion** |  |  | 0.704 |
| Yes | 177 (53.6) | 96 (51.9) |  |
| No | 153 (46.4) | 89 (48.1) |  |
| **Nerve invasion** |  |  | 0.266 |
| Yes | 205 (62.1) | 124 (67.0) |  |
| No | 125 (37.9) | 61 (33.0) |  |
| **HER2** |  |  | 0.887 |
| Positive | 17 (5.2) | 9 (4.9) |  |
| Negative | 313 (94.8) | 176 (95.1) |  |
| **PDL1** |  |  | 0.022 |
| Positive | 212 (64.2) | 137 (74.1) |  |
| Negative | 118 (35.8) | 48 (25.9) |  |
| **Chemotherapy** |  |  | 0.945 |
| Yes | 256 (77.6) | 144 (77.8) |  |
| No | 74 (22.4) | 41 (22.2) |  |
| **AGTR1** |  |  | 0.095 |
| Low | 171 (51.8) | 110 (59.5) |  |
| High | 159 (48.2) | 75 (40.5) |  |
| **DNER** |  |  | 0.293 |
| Low | 155 (47.0) | 78 (42.2) |  |
| High | 175 (53.0) | 107 (57.8) |  |
| **EPHA7** |  |  | 0.587 |
| Low | 183 (55.5) | 98 (53.0) |  |
| High | 147 (44.5) | 87 (47.0) |  |
| **SUSD5** |  |  | 0.317 |
| Low | 183 (55.5) | 111 (60.0) |  |
| High | 147 (44.5) | 74 (40.0) |  |

**Table S4. Clinical characteristics of the endoscopic biopsy specimen validation set cohort[n(%)]**

| **Clinical characteristic** | **Training cohort**  **(N=126)** |
| --- | --- |
| **Gender** |  |
| Male | 75 (59.5) |
| Female | 51 (40.5) |
| **Age(years)** |  |
| ≤65 | 70 (55.6) |
| ＞65 | 56 (44.4) |
| **T stage** |  |
| T2/T3 | 62 (49.2) |
| T4 | 64 (50.8) |
| **N stage** |  |
| N0 | 40 (31.7) |
| N+ | 86 (68.3) |
| **Primary site** |  |
| Up 1/3 | 40 (31.7) |
| Middle 1/3 | 37 (29.4) |
| Lower 1/3 | 49 (38.9) |
| **Tumor size(cm)** |  |
| ≤5 | 43 (34.1) |
| ＞5 | 83 (65.9) |
| **Histology** |  |
| None/Low | 15 (11.9) |
| High/Median | 111 (88.1) |
| **Lauren** |  |
| Diffuse/Mix type | 85 (67.5) |
| Intestinal type | 41 (32.5) |
| **Vascular invasion** |  |
| Yes | 63 (50.0) |
| No | 63 (50.0) |
| **Nerve invasion** |  |
| Yes | 83 (65.9) |
| No | 43 (34.1) |
| **HER2** |  |
| Positive | 6 (4.8) |
| Negative | 120 (95.2) |
| **PDL1** |  |
| Positive | 70 (55.6) |
| Negative | 56 (44.4) |
| **Chemotherapy** |  |
| Yes | 33 (26.2) |
| No | 93 (73.8) |
| **ATGR1** |  |
| Low | 68 (54.0) |
| High | 58 (46.0) |
| **DNER** |  |
| Low | 54 (42.9) |
| High | 72 (57.1) |
| **EPHA7** |  |
| Low | 69 (54.8) |
| High | 57 (45.2) |
| **SUSD5** |  |
| Low | 75 (59.5) |
| High | 51 (40.5) |

**Table S5. Clinical characteristics of peripheral blood specimen cohorts in training set and validation set[n(%)]**

| **Clinical characteristic** | **Training cohort**  **(N=136)** | **Validation cohort**  **(N=105)** | **P value** |
| --- | --- | --- | --- |
| **Gender** |  |  | 0.431 |
| Male | 80 (58.8) | 67 (63.8) |  |
| Female | 56 (41.2) | 38 (36.2) |  |
| **Age(years)** |  |  | 0.432 |
| ≤65 | 73 (53.7) | 51 (48.6) |  |
| ＞65 | 63 (46.3) | 54 (51.4) |  |
| **T stage** |  |  | 0.870 |
| T2/T3 | 31 (22.8) | 23 (21.9) |  |
| T4 | 105 (77.2) | 82 (78.1) |  |
| **N stage** |  |  | 0.550 |
| N0 | 37 (27.2) | 25 (23.8) |  |
| N+ | 99 (72.8) | 80 (76.2) |  |
| **Primary site** |  |  | 0.819 |
| Up 1/3 | 44 (32.4) | 36 (34.3) |  |
| Middle 1/3 | 40 (29.4) | 33 (31.4) |  |
| Lower 1/3 | 52 (38.2) | 36 (34.3) |  |
| **Tumor size(cm)** |  |  | 0.885 |
| ≤5 | 48 (35.3) | 38 (36.2) |  |
| ＞5 | 88 (64.7) | 67 (63.8) |  |
| **Histology** |  |  | 0.753 |
| None/Low | 120 (88.2) | 94 (89.5) |  |
| High/Median | 16 (11.8) | 11 (10.5) |  |
| **Lauren** |  |  | 0.294 |
| Diffuse/Mix type | 93 (68.4) | 65 (61.9) |  |
| Intestinal type | 43 (31.6) | 40 (38.1) |  |
| **Vascular invasion** |  |  | 0.826 |
| Yes | 68 (50.0) | 54 (51.4) |  |
| No | 68 (50.0) | 51 (48.6) |  |
| **Nerve invasion** |  |  | 0.455 |
| Yes | 83 (61.0) | 69 (65.7) |  |
| No | 53 (39.0) | 36 (34.3) |  |
| **HER2** |  |  | 0.773 |
| Positive | 9 (6.6) | 6 (5.7) |  |
| Negative | 127 (93.4) | 99 (94.3) |  |
| **PDL1** |  |  | 0.730 |
| Positive | 39 (28.7) | 28 (26.7) |  |
| Negative | 97 (71.3) | 77 (73.3) |  |
| **Chemotherapy** |  |  | 0.151 |
| Yes | 95 (69.9) | 82 (78.1) |  |
| No | 41 (30.1) | 23 (21.9) |  |
| **AGTR1** |  |  | 0.187 |
| Low | 70 (51.5) | 63 (60.0) |  |
| High | 66 (48.5) | 42 (40.0) |  |
| **DNER** |  |  | 0.075 |
| Low | 62 (45.6) | 60 (57.1) |  |
| High | 74 (54.4) | 45 (42.9) |  |
| **EPHA7** |  |  | 0.723 |
| Low | 72 (52.9) | 58 (55.2) |  |
| High | 64 (47.1) | 47 (44.8) |  |
| **SUSD5** |  |  | 0.963 |
| Low | 82 (60.3) | 63 (60.0) |  |
| High | 54 (39.7) | 42 (40.0) |  |

**Table S6. Primer sequences and qPCR conditions for the four genes**

| Name | Sequence |
| --- | --- |
| SUSD5_F | GACCGCCTTCACCTTGCTA |
| SUSD5_R | TGTCCGGGAATCCTCCATGA |
| EPHA7_F | CTAAACGTGGAGCAGCCGAT |
| EPHA7_R | CATGGTGCATGAGCAGGTTT |
| AGTR1_F | CGGGGCGCGGGTTTG |
| AGTR1_R | TCAAATACACCTGGTGCCGA |
| DNER_F | GCCGAAAACAGGGCAGAAAG |
| DNER_R | CACCCGCAGAGCTGTTAGAA |
| F:Forward | R:Reverse |
|  |  |
| The conditions for RNA extraction | Take part of the tissue and add 1ml TRIzol reagent, place it on ice and grind thoroughly. Subsequently adding 1/5 volume of chloroform to the mixture, mix by inverting upside down, and place the sample on ice for 5 minutes. Centrifuge at 12.000 rpm at high speed, 4℃ for 15 minutes. After l5 minutes, absorb the supernatant and add an equal volume of isopropyl alcohol. Mix by inverting and let stand on ice for 10 minutes. Centrifuge again at high speed, 12000 rpm, 4℃, for 10 minutes. After discarding the supernatant, add 400 ul of 75% ethanol, centrifuge at 9000 rpm, 4℃ for 5 minutes, Discard the supernatant and after the precipitate is dried, add an appropriate amount of DEPC water to dissolve the precipitate. |
|  |  |
| The PCR conditions | First set the pre-denaturation program to 95℃, 10 minutes. Afterwards, 40 cycles of reaction were carried out, with each cycle ending at 95℃ for 15 seconds for denaturation, 58℃ for 30 seconds for annealing, and 72℃ for 30 seconds for extension. Finally, set the dissolution curve program to 95℃, terminate the extension in 15 seconds, maintain the system temperature at 60℃ for 1 min. And finally increase the temperature by 0.3℃ per unit time to 95℃ for 15s. |

**Table S7. Multifactor logistic regression analysis of four candidate mRNAs that influence the recurrence and metastasis of LAGC patients after radical surgery**

| **Surgical resection specimens--training cohort** | | | |
| --- | --- | --- | --- |
| **mRNA** | **OR** | **95%CI** | **P value** |
| AGTR1 (High vs. Low) | 5.075 | 2.973-8.662 | ＜0.001 |
| DNER (High vs. Low) | 3.267 | 1.901-5.616 | ＜0.001 |
| EPHA7 (High vs. Low) | 2.068 | 1.212-3.529 | 0.008 |
| SUSD5 (High vs. Low) | 4.569 | 2.667-7.828 | ＜0.001 |
| **Surgical resection specimens--validation cohort** | | | |
| **mRNA** | **OR** | **95%CI** | **P value** |
| AGTR1 (High vs. Low) | 3.548 | 1.723-7.308 | 0.001 |
| DNER (High vs. Low) | 3.595 | 1.685-7.667 | 0.001 |
| EPHA7 (High vs. Low) | 4.815 | 2.323-9.980 | ＜0.001 |
| SUSD5 (High vs. Low) | 3.973 | 1.864-8.471 | ＜0.001 |
| **Gastroscopy biopsy specimens--validation cohort** | | | |
| **mRNA** | **OR** | **95%CI** | **P value** |
| AGTR1 (High vs. Low) | 4.805 | 1.991-12.082 | 0.001 |
| DNER (High vs. Low) | 3.328 | 1.248-8.871 | 0.016 |
| EPHA7 (High vs. Low) | 3.320 | 1.358-8.116 | 0.009 |
| SUSD5 (High vs. Low) | 4.146 | 1.662-10.340 | 0.002 |
| **Peripheral blood specimens--training cohort** | | | |
| **mRNA** | **OR** | **95%CI** | **P value** |
| AGTR1 (High vs. Low) | 4.956 | 2.176-11.289 | ＜0.001 |
| DNER (High vs. Low) | 3.800 | 1.644-8.784 | 0.002 |
| EPHA7 (High vs. Low) | 2.356 | 1.034-5.370 | 0.042 |
| SUSD5 (High vs. Low) | 3.748 | 1.625-8.643 | 0.002 |
| **Peripheral blood specimens--validation cohort** | | | |
| **mRNA** | **OR** | **95%CI** | **P value** |
| AGTR1 (High vs. Low) | 2.864 | 1.078-7.606 | 0.035 |
| DNER (High vs. Low) | 5.329 | 1.143-8.983 | 0.027 |
| EPHA7 (High vs. Low) | 9.959 | 1.752-12.174 | 0.002 |
| SUSD5 (High vs. Low) | 3.539 | 1.405-10.938 | 0.009 |

**Table S8. Multifactor logistic regression analysis that affects the occurrence of recurrence and metastasis in patients with LAGC after radical surgery**

| **Surgical resection specimens--training cohort** | | | |
| --- | --- | --- | --- |
| **Variables** | **OR** | **95%CI** | **P value** |
| TNM stage (III vs. I/II) | 2.136 | 1.330-3.430 | 0.002 |
| Nerve invasion (Yes vs. No) | 2.280 | 1.209-4.302 | 0.011 |
| Chemotherapy (No vs. Yes) | 2.577 | 1.176-5.648 | 0.018 |
| 4-mRNA panel (High vs. Low) | 8.466 | 4.749-15.095 | ＜0.001 |
| **Surgical resection specimens--validation cohort** | | | |
| **Variables** | **OR** | **95%CI** | **P value** |
| TNM stage (III vs. I/II) | 3.490 | 1.529-7.966 | 0.003 |
| Nerve invasion (Yes vs. No) | 5.218 | 1.662-16.385 | 0.005 |
| Chemotherapy (No vs. Yes) | 4.998 | 1.302-19.191 | 0.019 |
| 4-mRNA panel (High vs. Low) | 16.947 | 6.597-43.534 | ＜0.001 |
| **Gastroscopy biopsy specimens--validation cohort** | | | |
| **Variables** | **OR** | **95%CI** | **P value** |
| TNM stage (III vs. I/II) | 5.096 | 1.636-15.875 | 0.005 |
| Nerve invasion (Yes vs. No) | 4.721 | 1.075-20.734 | 0.040 |
| Chemotherapy (No vs. Yes) | 12.696 | 2.145-75.150 | 0.005 |
| 4-mRNA panel (High vs. Low) | 21.913 | 5.984-80.252 | ＜0.001 |
| **Peripheral blood specimens--training cohort** | | | |
| **Variables** | **OR** | **95%CI** | **P value** |
| TNM stage (III vs. I/II) | 6.741 | 1.938-23.450 | 0.003 |
| Nerve invasion (Yes vs. No) | 6.038 | 1.361-26.783 | 0.018 |
| Chemotherapy (No vs. Yes) | 12.332 | 2.087-72.857 | 0.006 |
| 4-mRNA panel (High vs. Low) | 25.787 | 6.845-97.151 | ＜0.001 |
| **Peripheral blood specimens--validation cohort** | | | |
| **Variables** | **OR** | **95%CI** | **P value** |
| TNM stage (III vs. I/II) | 8.769 | 2.001-38.431 | 0.004 |
| Nerve invasion (Yes vs. No) | 7.873 | 1.160-53.419 | 0.035 |
| Chemotherapy (No vs. Yes) | 23.791 | 2.956-191.499 | 0.003 |
| 4-mRNA panel (High vs. Low) | 46.554 | 7.609-284.822 | ＜0.001 |

**Table S9. Comparison of performance indicators of different models in predicting postoperative recurrence of gastric cancer patients in tissue specimens**

| **Variable** | **AUC** | **Accuracy** | **Sensitivity** | **Specificity** | **PPV** | **NPV** | **PLR** | **NLR** | **F1 score** |
| --- | --- | --- | --- | --- | --- | --- | --- | --- | --- |
| **Training set** |  |  |  |  |  |  |  |  |  |
| Clinical features | 0.745 | 0.656 | 0.632 | 0.677 | 0.603 | 0.704 | 1.959 | 0.543 | 0.617 |
| 4-mRNA panel | 0.827 | 0.739 | 0.806 | 0.688 | 0.667 | 0.821 | 2.583 | 0.283 | 0.730 |
| Risk-stratification model | 0.864 | 0.761 | 0.743 | 0.774 | 0.718 | 0.796 | 3.291 | 0.332 | 0.730 |
| **Validation set** |  |  |  |  |  |  |  |  |  |
| Clinical features | 0.769 | 0.686 | 0.662 | 0.703 | 0.598 | 0.757 | 2.227 | 0.481 | 0.628 |
| 4-mRNA panel | 0.822 | 0.778 | 0.743 | 0.802 | 0.714 | 0.824 | 3.750 | 0.320 | 0.728 |
| Risk-stratification model | 0.919 | 0.821 | 0.716 | 0.892 | 0.815 | 0.825 | 6.625 | 0.318 | 0.763 |

Note: AUC, area under the curve; PPV, Positive Predictive Value; NVP, Negative Predictive Value; PLR, Positive Likelihood Ratio; NLR, Negative Likelihood Ratio.

**Table S10. Comparison of performance indicators of different models in predicting postoperative recurrence in patients with gastric cancer in different subgroup analysis**

| **Variable** | **AUC** | **Accuracy** | **Sensitivity** | **Specificity** | **PPV** | **NPV** | **PLR** | **NLR** | **F1 score** |
| --- | --- | --- | --- | --- | --- | --- | --- | --- | --- |
| **TNM stage I-II** |  |  |  |  |  |  |  |  |  |
| Clinical features | 0.780 | 0.770 | 0.236 | 0.938 | 0.542 | 0.797 | 3.782 | 0.815 | 0.329 |
| 4-mRNA panel | 0.850 | 0.857 | 0.618 | 0.932 | 0.739 | 0.886 | 9.067 | 0.410 | 0.673 |
| Risk-stratification model | 0.889 | 0.874 | 0.600 | 0.960 | 0.825 | 0.885 | 15.086 | 0.417 | 0.695 |
| **TNM stage III** |  |  |  |  |  |  |  |  |  |
| Clinical features | 0.657 | 0.648 | 0.846 | 0.380 | 0.648 | 0.648 | 1.366 | 0.403 | 0.734 |
| 4-mRNA panel | 0.789 | 0.778 | 0.743 | 0.802 | 0.714 | 0.824 | 3.750 | 0.320 | 0.728 |
| Risk-stratification model | 0.830 | 0.782 | 0.804 | 0.752 | 0.814 | 0.740 | 3.242 | 0.261 | 0.809 |
| **HER2-positive** |  |  |  |  |  |  |  |  |  |
| Clinical features | 0.834 | 0.760 | 1.000 | 0.571 | 0.647 | 1.000 | 2.333 | 0.000 | 0.786 |
| 4-mRNA panel | 0.724 | 0.680 | 0.273 | 1.000 | 1.000 | 0.636 | N/A | 0.727 | 0.429 |
| Risk-stratification model | 0.844 | 0.760 | 1.000 | 0.571 | 0.647 | 1.000 | 2.333 | 0.000 | 0.786 |
| **HER2-negative** |  |  |  |  |  |  |  |  |  |
| Clinical features | 0.770 | 0.669 | 0.633 | 0.695 | 0.604 | 0.721 | 2.075 | 0.528 | 0.618 |
| 4-mRNA panel | 0.827 | 0.742 | 0.763 | 0.727 | 0.672 | 0.807 | 2.795 | 0.326 | 0.715 |
| Risk-stratification model | 0.883 | 0.757 | 0.710 | 0.791 | 0.714 | 0.788 | 3.394 | 0.367 | 0.712 |
| **PDL1-positive** |  |  |  |  |  |  |  |  |  |
| Clinical features | 0.791 | 0.684 | 0.708 | 0.667 | 0.626 | 0.743 | 2.123 | 0.438 | 0.665 |
| 4-mRNA panel | 0.833 | 0.771 | 0.688 | 0.836 | 0.768 | 0.773 | 4.194 | 0.373 | 0.726 |
| Risk-stratification model | 0.893 | 0.765 | 0.695 | 0.821 | 0.754 | 0.773 | 3.871 | 0.372 | 0.723 |
| **PDL1-negative** |  |  |  |  |  |  |  |  |  |
| Clinical features | 0.695 | 0.693 | 0.531 | 0.794 | 0.618 | 0.730 | 2.580 | 0.590 | 0.571 |
| 4-mRNA panel | 0.799 | 0.675 | 0.781 | 0.608 | 0.556 | 0.816 | 1.992 | 0.360 | 0.649 |
| Risk-stratification model | 0.841 | 0.753 | 0.656 | 0.814 | 0.689 | 0.790 | 3.523 | 0.422 | 0.672 |

Note: AUC, area under the curve; PPV, Positive Predictive Value; NVP, Negative Predictive Value; PLR, Positive Likelihood Ratio; NLR, Negative Likelihood Ratio.

**Table S11. Comparison of performance indicators of different models in predicting postoperative recurrence of gastric cancer patients in gastroscopy biopsy specimens**

| **Variable** | **AUC** | **Accuracy** | **Sensitivity** | **Specificity** | **PPV** | **NPV** | **PLR** | **NLR** | **F1 score** |
| --- | --- | --- | --- | --- | --- | --- | --- | --- | --- |
| Clinical features | 0.793 | 0.730 | 0.647 | 0.787 | 0.673 | 0.766 | 3.033 | 0.448 | 0.660 |
| 4-mRNA panel | 0.838 | 0.802 | 0.686 | 0.880 | 0.795 | 0.805 | 5.719 | 0.357 | 0.737 |
| Risk-stratification model | 0.906 | 0.825 | 0.725 | 0.893 | 0.822 | 0.827 | 6.801 | 0.307 | 0.771 |

Note: AUC, area under the curve; PPV, Positive Predictive Value; NVP, Negative Predictive Value; PLR, Positive Likelihood Ratio; NLR, Negative Likelihood Ratio.

**Table S12. Comparison of performance indicators of different models in predicting postoperative recurrence in patients with gastric cancer in peripheral blood samples**

| **Variable** | **AUC** | **Accuracy** | **Sensitivity** | **Specificity** | **PPV** | **NPV** | **PLR** | **NLR** | **F1 score** |
| --- | --- | --- | --- | --- | --- | --- | --- | --- | --- |
| **Training set** |  |  |  |  |  |  |  |  |  |
| Clinical features | 0.797 | 0.735 | 0.726 | 0.743 | 0.703 | 0.764 | 2.827 | 0.369 | 0.714 |
| 4-mRNA panel | 0.824 | 0.750 | 0.855 | 0.662 | 0.679 | 0.845 | 2.530 | 0.219 | 0.757 |
| Risk-stratification model | 0.883 | 0.801 | 0.806 | 0.797 | 0.769 | 0.831 | 3.978 | 0.243 | 0.787 |
| **Validation set** |  |  |  |  |  |  |  |  |  |
| Clinical features | 0.872 | 0.743 | 0.579 | 0.836 | 0.667 | 0.778 | 3.526 | 0.504 | 0.620 |
| 4-mRNA panel | 0.824 | 0.762 | 0.763 | 0.761 | 0.644 | 0.850 | 3.196 | 0.311 | 0.699 |
| Risk-stratification model | 0.935 | 0.838 | 0.711 | 0.910 | 0.818 | 0.847 | 7.934 | 0.318 | 0.761 |

Note: AUC, area under the curve; PPV, Positive Predictive Value; NVP, Negative Predictive Value; PLR, Positive Likelihood Ratio; NLR, Negative Likelihood Ratio.

**Table S13. Comparison of performance indicators of different models in predicting postoperative recurrence in patients with gastric cancer in peripheral blood tumor marker negative specimens**

| **Variable** | **AUC** | **Accuracy** | **Sensitivity** | **Specificity** | **PPV** | **NPV** | **PLR** | **NLR** | **F1 score** |
| --- | --- | --- | --- | --- | --- | --- | --- | --- | --- |
| Clinical features | 0.680 | 0.680 | 0.619 | 0.724 | 0.619 | 0.724 | 2.244 | 0.526 | 0.619 |
| 4-mRNA panel | 0.838 | 0.802 | 0.686 | 0.880 | 0.795 | 0.805 | 5.719 | 0.357 | 0.737 |
| Risk-stratification model | 0.906 | 0.780 | 0.714 | 0.828 | 0.750 | 0.800 | 4.143 | 0.345 | 0.732 |

Note: AUC, area under the curve; PPV, Positive Predictive Value; NVP, Negative Predictive Value; PLR, Positive Likelihood Ratio; NLR, Negative Likelihood Ratio.

**Table S14. The siRNA sequences for the four genes**

| mRNA | siRNA sequences |
| --- | --- |
| AGTR1 | siAGTR1-1: GCAGTAGCCAGCAATTTGA |
|  | siAGTR1-2: ATAAGAAGGTTCAGATCCA |
| DNER | siDNER-1: GUGUGACCCCCCUUCAGGC |
|  | siDNER-2: GCAGUACGUGGGUACUUUC |
| EPHA7 | siEPHA7-1: GGCUCAAGUGGAAAUCCUA |
|  | siEPHA7-2: CCAGCAGCUUCUAAUAAUA |
| SUSD5 | siSUSD5-1:GCUCUUAACUUCUUCUCAA |
|  | siSUSD5-2:CAGGAAGGUGCUCUAUGUA |
